# Synergistic Doping and Stabilization of Magnetically Tunable *Ln*Ti_3_(Sb,Sn)_4_ (*Ln*: Ce–Gd) Kagome Metals

**DOI:** 10.1021/jacs.6c06163

**Published:** 2026-07-23

**Authors:** Brenden R. Ortiz, Ramakanta Chapai, German D. Samolyuk, Milo Sprague, Arun K. Kumay, Hu Miao, Karolina Górnicka, Xiaoping Wang, Qiang Zhang, Madhab Neupane, David Parker, Jiaqiang Yan

**Affiliations:** † Materials Science and Technology Division, 560212 Oak Ridge National Laboratory, Oak Ridge, Tennessee 37831, United States; ‡ Department of Physics, 6041 Norfolk State University, Norfolk, Virginia 23504, United States; § Department of Physics, 6243 University of Central Florida, Orlando, Florida 32816, United States; ∥ Applied Physics and Mathematics Department, Advanced Materials Centre, Gdansk University of Technology, ul. Narutowicza 11/12, 80-233 Gdansk, Poland; ⊥ Neutron Scattering Division, 6146 Oak Ridge National Laboratory, Oak Ridge, Tennessee 37831, United States

## Abstract

Here, we present our synthesis and characterization of the *Ln*Ti_3_(Sb,Sn)_4_ (*Ln*: Ce, Pr, Nd, Sm, Gd) family of cleavable kagome metals. While these materials are isostructural to the *Ln*Ti_3_Bi_4_ family, they only form as (Sb,Sn) solid-solutions with no corresponding *Ln*Ti_3_Sb_4_ or *Ln*Ti_3_Sn_4_ phases. We use a combination of first-principles density functional theory (DFT) and crystal orbital Hamilton population (COHP) calculations to show that (Sb,Sn) alloying has a stabilizing effect on the structure by adjusting the Fermi level, filling bonding states, depopulating antibonding states, and adjusting the density of states (DOS) toward local minima, an effect we call “synergistic doping.” Through a detailed characterization of the SmTi_3_(Sb,Sn)_4_ series, we further demonstrate that the tunable Fermi level has a profound effect on the physical properties. We observe multiple magnetic phases that stem from a competition between antiferromagnetic (AFM) and ferromagnetic-like (FM) ground states. Furthermore, the (Sb,Sn) ratio allows us to tune from direct AFM-FM competition toward a complex admixture with properties reminiscent of the recently discovered TbTi_3_Bi_4_. Ultimately, our work demonstrates how the concept of synergistic doping provides a means to stabilize new structures while developing systems with intrinsically tunable chemical, magnetic, and electronic properties.

## Graphical abstract

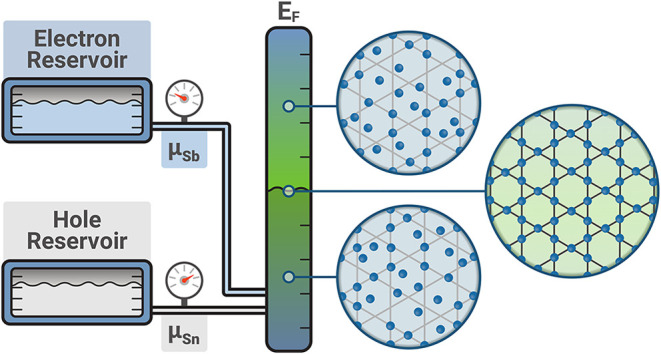

## Introduction

Research into kagome intermetallics continues to represent a key intersection between fundamental condensed matter physics and solid-state chemistry. In particular, the search for materials that host complex magnetic and electronic instabilities has drawn significant attention to the distinctive band structure associated with kagome metals, which produces an electronic structure replete with Dirac points, flat bands, and van Hove singularities.^1–3^ Our discovery of the *A*V_3_Sb_5_ (*A*: K, Rb, Cs) kagome metals, which host a charge density wave and a superconducting ground state,^4,5^ exemplified the latent potential of the kagome motif. Since then, an extensive body of research has emerged investigating emergent topological, electronic, and magnetic instabilities (e.g., charge density waves and superconductivity) within kagome metals.^6–12^

Unfortunately, the chemical flexibility of the *AM*_3_ *X*_5_ family is limited. Since the original discovery, only three other materials have been discovered: CsCr_3_Sb_5_,^13^ CsTi_3_Bi_5_,^14^ and RbTi_3_Bi_5_.^14^ An alternative set of charge density wave materials emerged in the CoSn and related families (e.g., *AM*_6_ *X*_6_), with discoveries of density-wave behavior exhibited in LuNb_6_Sn_6_,^15^ ScV_6_Sn_6_,^16^ and FeGe.^17^ However, unlike the electronically driven instabilities intrinsic to the *AM*_3_ *X*_5_ family, the CoSn-based materials are primarily driven by steric effects. Further, only a small percentage of the known compounds exhibit density-wave instabilities.^18–29^ As such, there remains a strong impetus for the discovery of more kagome intermetallics that host complex electronic and magnetic instabilities.

The *AM*_3_ *X*_4_ family is another rapidly developing class of kagome metals, with both the antimonide *Ln*V_3_Sb_4_ (*Ln*: Eu^2+^, Yb^2+^)^30^ and bismuthide *Ln*Ti_3_Bi_4_ (*Ln*: La···Tb^3+^, Eu^2+^, Yb^2+^) families reported recently.^30–35^ This family exhibits zigzag chains of *A* and kagome nets comprised of *M*. A wide range of electropositive alkali-earth and rare-earth elements are able to substitute on *A*, yielding a wide range of magnetic properties. Recently, TbTi_3_Bi_4_,^36^ GdTi_3_Bi_4_,^37^ and CeTi_3_Bi_4_ ^38^ have been reported to host spin- and/or charge density anomalies. The availability of large, cleavable single crystals has also enabled many advanced characterization techniques like angle-resolved photoemission spectroscopy (ARPES),^39–47^ scanning-tunneling microscopy (STM),^37,44^ and other complementary techniques (e.g., magnetic force microscopy, torque magnetometry, and neutron diffraction).^44,48,49^ A wide array of complex magnetotransport phenomena^36,50–52^ have also been observed, including a record-large anomalous Hall effect (AHE) in TbTi_3_Bi_4_.^36^

This work focuses on the synthesis and characterization of an analogous series of materials, the *Ln*Ti_3_(Sb,Sn)_4_ (*Ln*: Ce–Gd) metals. These materials exist only as a (Sb,Sn) solid solution between the hypothetical *Ln*Ti_3_Sn_4_ and *Ln*Ti_3_Sb_4_ parent structures. We first explore the stabilizing effect of the (Sb,Sn) alloying using a combination of density functional theory (DFT) and crystal orbital Hamilton population (COHP) calculations. We demonstrate that the chemical and steric similarity of the (Sb,Sn) pair imbues the system with a natural charge degree of freedom, allowing systematic adjustment of the Fermi level and optimization of bonding/antibonding interactions—an effect we call “synergistic doping.” The variable Fermi level also manifests as tunable magnetism, which we demonstrate through a detailed investigation of the SmTi_3_(Sb,Sn)_4_ physical properties. We show that the system exhibits both ferromagnetic (FM) and antiferromagnetic (AFM) signatures, and that the (Sb,Sn) ratio controls the blending and dominance of the interactions. We also briefly comment on the (Sb,Sn) control and analogous properties in the other rare-earth (*Ln*: Ce, Pr, Nd, Gd) compounds. Our results ultimately show how the (Sb,Sn) synergistic dopants stabilize the *Ln*Ti_3_(Sb,Sn)_4_ crystal structure, control the magnetic ground state, and provide a new synthetic strategy for the discovery of complex intermetallic compounds.

## Experimental Methods

### Synthesis

*Ln*Ti_3_(Sb,Sn)_4_ (*Ln*: Ce–Gd) single crystals were grown from alloys of (Sb,Sn) using the self-flux method. Rare-earth metal pieces (Ames 99.9+%) were placed with Ti powder (Alfa 99.9%), Sb shot (Alfa 99.999%), and Sn shot (Alfa 99.9%) into 2 mL Canfield crucibles fitted with a catch crucible and a porous frit.^53^ The ratio of *Ln* and Ti was fixed relative to the total amount of (Sb,Sn) flux such that the total composition adhered to *Ln*_0.25_Ti_0.75_Sb_*x*_Sn_10–*x*_. The range of (Sb,Sn) compositions that yielded the desired *Ln*Ti_3_(Sb,Sn)_4_ phase is discussed later.

The filled crucibles were sealed under approximately 0.7 atm of argon gas in fused silica ampules. Samples were heated to 1050 °C at a rate of 200 °C/h and homogenized at 1050 °C for 12 h. The vast majority of samples were cooled to 750 °C at a rate of 2 °C/h. However, we observed that Sn-rich compositions often required further cooling to 650 °C to form the desired phase. Excess (Sb,Sn) flux was removed through centrifugation at either 750 or 650 °C.

Single crystals of *Ln*Ti_3_(Sb,Sn)_4_ are silver-colored hexagonal plates with a metallic luster. Sample sizes commonly range from 1–10 mm, depending on the rare-earth species and the specific ratio of (Sb,Sn). Unlike the structurally analogous *Ln*Ti_3_Bi_4_ compounds, *Ln*Ti_3_(Sb,Sn)_4_ are completely stable in air and atmospheric moisture. Samples have remained untarnished after 6–12 months, unlike the *Ln*Ti_3_Bi_4_ compounds, which tarnish and spall within a few hours in air. *Ln*Ti_3_(Sb,Sn)_4_ crystals retain their easy (00L) cleavage plane, and can be readily cleaved with a sharp blade. Cleaved surfaces can be further exfoliated using adhesive tape.

### Bulk Characterization

Structural characterization of the *Ln*Ti_3_(Sb,Sn)_4_ series was performed through single-crystal X-ray diffraction (SCXRD) on a Bruker D8 Quest single-crystal diffractometer with a graphite monochromator and Mo Kα radiation (λ = 0.71073 Å). As noted in previous manuscripts,^31–33^ SCXRD is a challenging endeavor on these materials due to their large size, extremely soft mechanical properties, and exfoliable nature. To produce fragments for diffraction, we repeatedly exfoliated crystals using double-stick adhesive tape supported by glass microscope slides. The adhesive was subsequently dissolved using ethanol and acetone, and fragments were collected from the effluent. Fragments were transferred to oil and selected based on size, ensuring exfoliated fragments were not bent or warped. Flakes were then mounted on Kapton loops with Paratone oil for single-crystal X-ray diffraction (SCXRD). Face-indexed numerical absorption corrections were required to capture the extreme aspect ratio of the crystals, as the thicknesses were often 10–100 times thinner than the lateral dimensions. Data integration, reduction, and structure refinement were performed using the Bruker APEX4 software package. We performed additional (00L) facet scans on a Malvern Panalytical Empyrean powder X-ray diffractometer for an accurate determination of the *c* lattice parameter as a function of composition. The resulting scans were analyzed using the Pawley refinement method in TOPAS Academic V6 software.^54^ Due to the difficulty of resolving Sb and Sn in X-ray diffraction, we also performed preliminary single-crystal neutron diffraction measurements on Sb-rich crystals of NdTi_3_(Sb,Sn)_4_ (295 K). Measurements were performed on the TOPAZ beamline at the Spallation Neutron Source, Oak Ridge National Laboratory, and additional details can be found in the supplementary Tables S7 and S8.^55^

Elemental analysis of single crystals was carried out using a Hitachi TM3000 microscope equipped with a Bruker Quantax 70 EDS system. Multiple crystals (3–5) for each composition were mounted on carbon tape and cleaved to reveal pristine, flat surfaces before elemental analysis was performed (5–10 points per sample face). Results represent the average over several crystals and multiple locations to ensure sample homogeneity. To ensure the accuracy of the unstandardized SEM-EDS measurements, we also commissioned standardized wavelength-dispersive spectroscopy (WDS) using an electron microprobe (JEOL 8200) equipped with five tunable wavelength-dispersive spectrometers on samples of the Sb-rich and Sn-rich SmTi_3_(Sb,Sn)_4_. Three crystals of each composition were measured, with 50 measurements taken per exfoliated surface. The accelerating voltage was set to 20.0 kV, and the beam current was 50.0 nA. Elements were acquired using a PET crystal for Ti kα, Sn lα, Sb lα, and Sm lα. Pure standards were used to calibrate all elements except for Sm, for which SmF_3_ was used. Unknown and standard intensities were corrected for deadtime. Standard intensities were corrected for standard drift measured between the beginning and end of the run. Interference corrections were applied to Sn for interference by Sb and vice versa. The ZAF algorithm used was Armstrong/Love Scott.

Magnetization measurements (300–1.8 K) on crystals of *Ln*Ti_3_(Sb,Sn)_4_ were performed in a 7 T Quantum Design Magnetic Property Measurement System (MPMS3) SQUID magnetometer in vibrating-sample magnetometry (VSM) mode. Heat capacity measurements (300–1.8 K) were performed in a Quantum Design 9 T Dynacool Physical Property Measurement System (PPMS). Heat capacity measurements utilized the large-pulse (30% temperature rise) method, subsequently processed by both the dual-slope and single-slope methods. Electronic transport measurements were performed in a Quantum Design 12 T Dynacool PPMS equipped with the Electronic Transport Option (ETO). An alternating current of 10 mA and a frequency of 100 Hz was applied as the probe current for magnetoresistance measurements.

### Electronic Structure

Angle-resolved photoemission spectroscopy (ARPES) measurements in the main manuscript (and Figure S1) were carried out at the Advanced Light Source (ALS) at Lawrence Berkeley National Laboratory. Data were collected at beamline 10.0.1.1 using a Scienta R4000 hemispherical electron analyzer with an incident photon energy of 60 eV. The angular and total energy resolutions were set to 0.2° and 15 meV, respectively. High-quality single crystals were cut and mounted on copper posts using silver epoxy, with ceramic posts affixed to the samples. After loading into the main chamber, the samples were cooled and maintained under vacuum for several hours before cleaving. All subsequent measurements were performed at 20 K under ultrahigh vacuum conditions (<10^–10^ Torr). Supplementary ARPES (Figure S2) was performed at beamline 21-ID-1 of NSLS-II at Brookhaven National Lab (BNL) with an incident energy of 100 eV, linearly polarized photons, and 15 meV energy resolution.

DFT results utilized self-consistent methods^56^ to examine the electronic structure of SmTi_3_Sb_4_ and SmTi_3_Sn_4_, the hypothetical endmembers of the SmTi_3_(Sb,Sn)_4_ solid solution. The electronic exchange correlations were incorporated using the generalized gradient approximation (GGA) within the Perdew–Burke–Ernzerhof (PBE)^57^ parametrization. The plane-wave basis projector augmented-wave approach (PAW)^58^ as implemented in the Vienna Ab-initio Simulation Package (VASP)^59,60^ was used. We used a plane-wave energy cutoff of 450 eV and a 6 × 6 × 6 *k*-point mesh to perform the self-consistent calculations. We used the Sm^3+^ pseudopotential to capture electron–ion interaction for the Sm atoms, which is sufficient to describe the details of the band structure in the nonmagnetic state. Decomposed density of states calculations (shown in the Supporting Information, Figure S1) indicate that Sm states are approximately 1.5–2 eV below *E*_F_, suggesting that calculations near *E*_F_ are less reliant on the magnetic nature of the Sm ions and will serve as a good approximation for this work. For Ti, the pseudopotential used in these calculations treated the 3p semicore states as valence states. Both hypothetical SmTi_3_Sb_4_ and SmTi_3_Sn_4_ structures were relaxed independently with convergence criteria of <1 kbar for pressures, <10^–6^ eV for energies, and <10 meV/Å for interatomic forces.

For a chemical interpretation of the bonds within SmTi_3_(Sb,Sn)_4_, we employed crystal orbital Hamilton population (COHP) analysis. This represents the decomposition of the band structure over pair-projected orbitals^61,62^ and ultimately allows us to identify bonding and antibonding contributions for pairwise atomic interactions. The COHP calculations used our VASP results^63^ as inputs to the LOBSTER COHP package.^64^ Postprocessing of the LOBSTER results was performed using the LOPOSTER package.^65^

## Results and Discussion

### Synthesis and Crystal Structure

Initial attempts to prepare the *Ln*Ti_3_Sb_4_ analogues of the *Ln*Ti_3_Bi_4_ phases^31–35^ via Sb self-flux were unsuccessful. However, using an Sn-based flux produced the structurally analogous *Ln*Ti_3_(Sb,Sn)_4_ (*Ln*: Ce–Gd) series of alloys. An abbreviated representation of the crystal structure is shown in Figure 1(a). The structure has two sublattices of interest: (1) The *Ln*-based zigzag chains, and (2) the Ti-based kagome network, both highlighted in Figure 1(b). Compared to the corresponding bismuthide, the unit cell shrinks by approximately 7% (SmTi_3_Bi_4_:1500 Å^3^, Sb-rich SmTi_3_(Sb,Sn)_4_:1400 Å^3^), which is reflected in the associated Ti–Ti and *Ln*–*Ln* interatomic distances. Owing to the stronger Sb–Ti bonds, *Ln*Ti_3_(Sb,Sn)_4_ crystals are stable in air and atmospheric moisture for at least a year, a dramatic improvement over the air-sensitive (<1 h) bismuthides.

**Figure 1:**
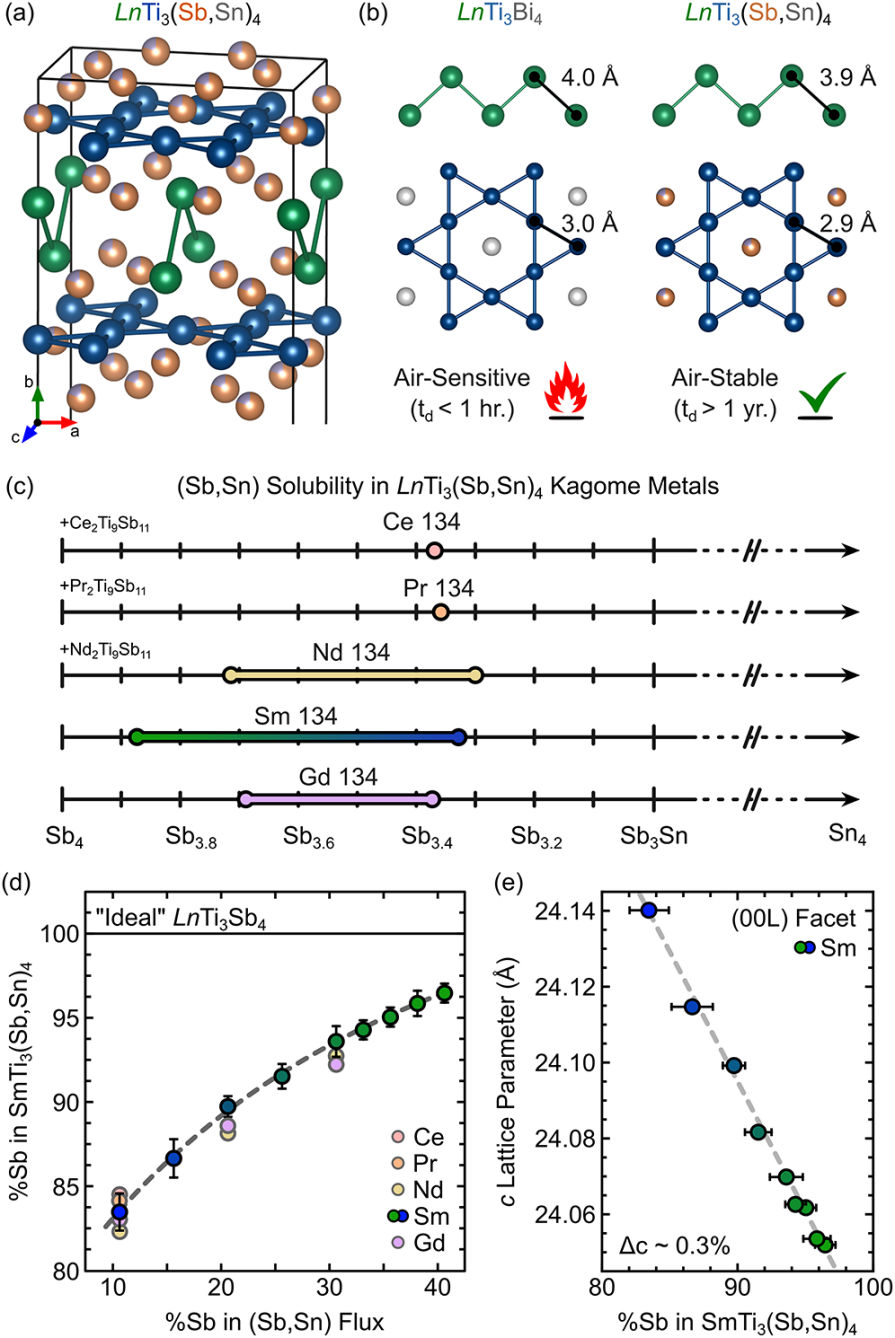
(a) Abbreviated view of the *Fmmm Ln*Ti_3_(Sb,Sn)_4_ crystal structure. (b) Schematic comparison of structural motifs in the *Ln*Ti_3_Bi_4_ and *Ln*Ti_3_(Sb,Sn)_4_ families. (c) (Sb,Sn) solubility ranges for each of the flux-grown *Ln*Ti_3_(Sb,Sn)_4_ single crystals. (d) Diagram showing the incorporation of (Sb,Sn) into the crystal structure depending on the nominal Sb percentage in the flux. (e) *c*-axis lattice parameter derived from 00L facet scans as a function of spectroscopic composition, demonstrating uniform incorporation of (Sb,Sn) in the host lattice.

The *Ln*Ti_3_(Sb,Sn)_4_ series contains a substantial amount of (Sb,Sn) mixing, and we were unable to isolate either the pure antimonide or the pure stannide using flux methods. A prior report from Bie et al. also notes their inability to form either SmTi_3_Sb_4_ or NdTi_3_Sb_4_ using solid-state methods,^66^ suggesting that these phases may not exist. Using (Sb,Sn) alloys as the flux, we prepared single crystals of *Ln*Ti_3_(Sb,Sn)_4_ for the extended series from *Ln*: (Ce–Gd), and further identified the solubility range under a variety of (Sb,Sn) ratios and synthetic conditions.

Figure 1(c) summarizes the range of accessible (Sb,Sn) alloys for *Ln*: (Ce–Gd). Compositions were measured using SEM-EDS, and error bars are derived from the standard deviation of independent measurements performed on 3–5 crystals of each composition with 5–10 data points per crystal. To check the accuracy of SEM-EDS, we performed additional standardized WDS measurements on the Sb-rich and Sn-rich termini of the SmTi_3_(Sb,Sn)_4_ solid solution. We find excellent agreement between SEM-EDS and WDS. For Sb-rich compositions, we find EDS: Sb_3.869(1)_Sn_0.14(1)_ and WDS: Sb_3.857(5)_Sn_0.143(2)_. For Sn-rich compositions, the corresponding values are EDS: Sb_3.34(1)_Sn_0.66(1)_ and WDS: Sb_3.327(15)_Sn_0.673(14)_. We will reference SEM-EDS compositions for the remainder of the paper.

Despite being grown in (Sb,Sn) alloys relatively rich in Sn, the resulting crystals are much closer to the hypothetical antimonide SmTi_3_Sb_4_ than they are to SmTi_3_Sn_4_. Figure 1(d), which shows the nominal (Sb,Sn) flux composition versus the actual (Sb,Sn) incorporation in the single crystals, further highlights this point. Even at 10% nominal Sb loading in the flux, the average (Sb,Sn) site occupancy is >80% Sb, though there are diminishing returns as more Sb is added to the flux. After 40–50% Sb, all series terminate with the crystallization of other ternary and quaternary phases. We have observed that altering the growth temperatures and the ratio of starting reagents (e.g., *Ln*_0.25_Ti_0.75_Sb_*x*_Sn_10–*x*_ vs *Ln*_0.25_Ti_0.75_Sb_2*x*_Sn_20–2*x*_) will change the correspondence between actual and nominal compositions, but will not enable further incorporation of Sb. We suspect that the reduced (Sb,Sn) solubility with Ce and Pr may be a consequence of the competing ternary antimonide *Ln*_2_Ti_9_Sb_11_ (*Ln*: La–Nd) we reported previously, though our tests of alternative flux compositions and temperature profiles were not exhaustive.^67^

Alloyed single crystals can often have challenges with chemical homogeneity because of the partitioning of different solutes as the flux cools. However, the (Sb,Sn) distribution in the *Ln*Ti_3_(Sb,Sn)_4_ series seems to be quite uniform. Our compositional measurements (EDS and WDS) show a low variation across macroscopic regions of the exfoliated crystals and at different depths within the crystals. We also performed (00L) facet scans to probe for peak splitting caused by the averaging of multiple unit cell volumes but observed none. Figure 1(e) plots the *c*-axis lattice parameter extracted from the (00L) facet scans as a function of the measured (Sb,Sn) composition. The strong linearity of Figure 1(e) is further evidence of the homogeneity of these samples. Since Sb and Sn are similarly sized and exhibit similar intermetallic chemistry, resolving the 0.3% change in the *c*-axis across the entire (Sb,Sn) series would not be possible if the samples were inhomogeneous. This effect is over an order of magnitude weaker than the volumetric change across the rare-earth series (3.5% between CeTi_3_(Sb,Sn)_4_ and GdTi_3_(Sb,Sn)_4_). The corresponding plot of the cell volume versus the nine-coordinate Shannon radius can be found in the Supporting Information (Figure S4).

Despite the apparent compositional homogeneity, we have previously observed preferential occupation of Sn in Sn-doped CsV_3_Sb_5–*x*_Sn_*x*_,^68^ which served as the impetus for a preliminary neutron scattering experiment on Sn-rich NdTi_3_Sb_3.3_Sn_0.7_. While improved over X-ray methods, neutron diffraction is also limited by the weak difference in coherent neutron scattering lengths between Sb and Sn. Our data indicate that the crystals are well-described by the *AM*_3_ *X*_4_ structure. Using NdTi_3_Sb_4_ as the model yields R1 = 3.67, and wR2 = 11.09. Including Sn in the model (NdTi_3_(Sb,Sn)_4_) yields small but consistent decreases in the R-factor (3.60–3.62) and wR2 = (10.84–10.94), depending on the specifics of occupancy distribution. We see no evidence for a (Sb,Sn)-based structural superlattice, but given the weak change in R-factors, we are hesitant to comment on any potential Sn site specificity. However, we believe it is important that CIF files accurately depict these compounds as (Sb,Sn) alloys. The published CIF files and the supplementary summary Tables (S1–S6) assign (Sb,Sn) uniformly across all sites, using the average compositions determined by WDS and SEM-EDS. Details and methodology, including refinement results for single-crystal neutron diffraction, are shown in Supporting Information (Tables S7 and S8). Determination of potential preferential occupation is progressing as a part of magnetic structure determination, but is beyond the scope of this current work.

For the remainder of this manuscript, we will focus on the SmTi_3_(Sb,Sn)_4_ series because it shows the largest variation in (Sb,Sn) incorporation and exhibits strong magnetic anisotropy (easy *c*-axis), facilitating sample alignment. Throughout the paper, we will refer to the Sb-rich terminus of the solid solution (WDS: SmTi_3_Sb_3.86_Sn_0.14_) as Sb-rich SmTi_3_(Sb,Sn)_4_, and it will be colored green in all subsequent plots. Similarly, the (relatively) Sn-rich composition (WDS: SmTi_3_Sb_3.33_Sn_0.67_) will be referred to as Sn-rich SmTi_3_(Sb,Sn)_4_ and will be colored blue. We have included a brief discussion of the other rare-earth LnTi_3_(Sb,Sn)_4_ compounds (Ln: Ce, Pr, Nd, Gd) in parallel to our discussion of SmTi_3_(Sb,Sn)_4_, though the bulk of the discussion and characterization data is relegated to the Supporting Information (Figures S5–S8).

### “Synergistic Doping” and Stability of *Ln*Ti_3_(Sb,Sn)_4_

Both our work and work from Bie et al.^66^ suggest that the *Ln*Ti_3_(Sb,Sn)_4_ compounds exist only as an alloy between the hypothetical compounds *Ln*Ti_3_Sb_4_ and *Ln*Ti_3_Sn_4_. We hypothesize that the structure may be stabilized by a certain electron count, and that the chemical similarity (radii, electronegativity, and bonding tendencies) of Sb and Sn allows the system to naturally equilibrate at the electron count that stabilizes the structure. For brevity, we are calling this effect—imbuing a system with chemically similar alloying agents to enable a natural charge degree of freedom and stabilize a structure—“synergistic doping.” In this section, we will combine experimental angle-resolved photoemission spectroscopy (ARPES) and first-principles density functional theory (DFT) to examine whether synergistic doping can explain the stabilization of the *Ln*Ti_3_(Sb,Sn)_4_ series. As before, we will focus on SmTi_3_(Sb,Sn)_4_.

First, we establish that (Sb,Sn) enables tunability of the Fermi level (*E*_F_) in SmTi_3_(Sb,Sn)_4_ without drastically changing the electronic structure. To quantify changes in *E*_F_ induced by alloying, we performed experimental ARPES measurements on Sn-rich (SmTi_3_Sb_3.34_Sn_0.66_) and Sb-rich (SmTi_3_Sb_3.86_Sn_0.14_) single crystals. These results are shown in Figure 2(a,b), respectively. To determine the doping shift of *E*_F_, we examine the intersection of a series of dispersive bands crossing through Γ and intersecting a concave-down band. The bands have been highlighted in the figure with a light gray trace, and the intersection (*E*_cross_) has been marked with a dashed black line. Subtracting *E*_cross,Sn_ (−0.51 eV) from *E*_cross,Sb_ (−0.64 eV) yields a shift of 130 meV. Normalizing this shift to the (per atom) concentration difference between SmTi_3_Sb_3.33_Sn_0.67_ and SmTi_3_Sb_3.86_Sn_0.14_, we observe a shift of 250 meV per (Sb,Sn) substitution.

**Figure 2:**
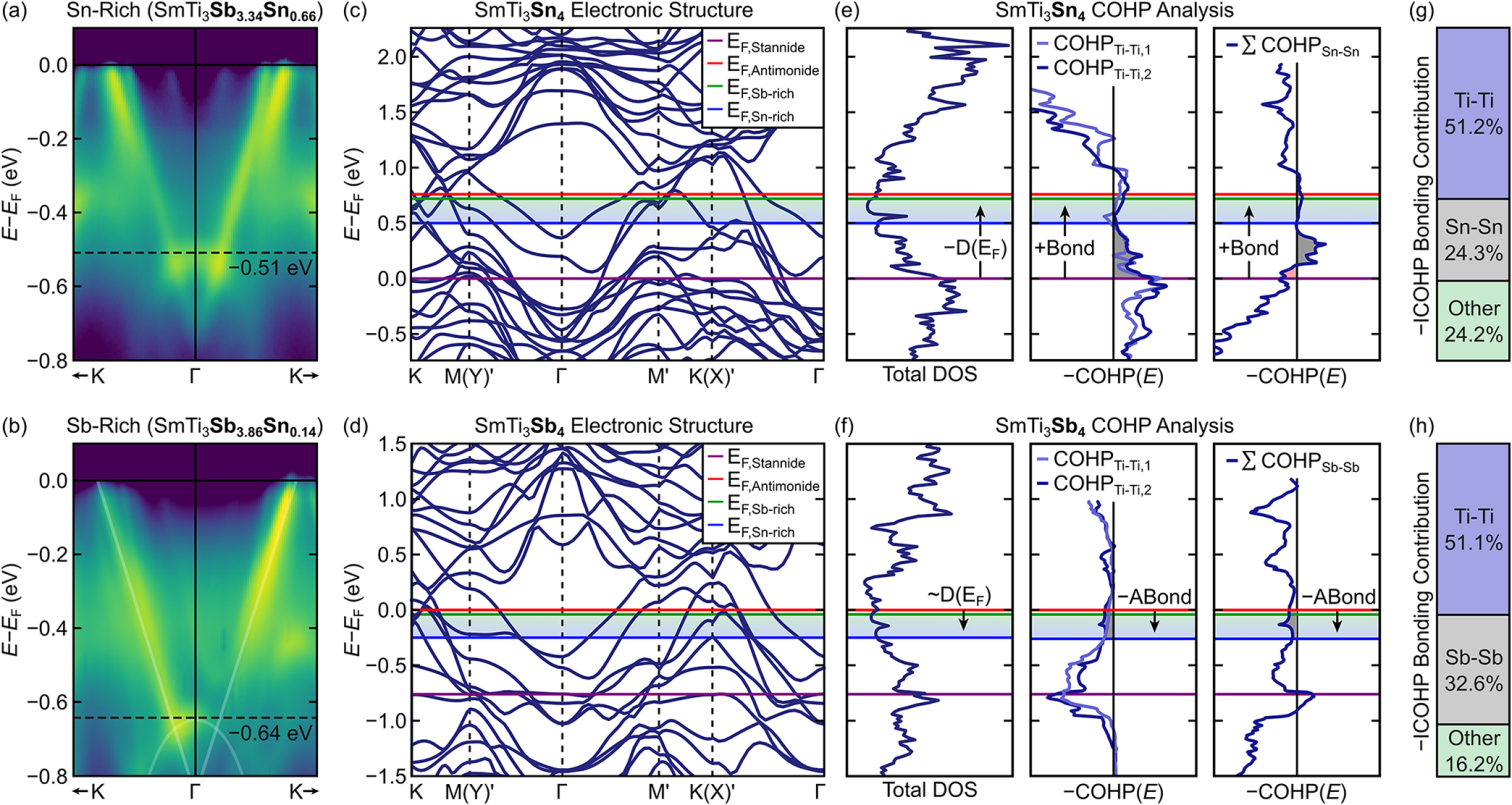
(a, b) ARPES data for Sn-rich (a) and Sb-rich (b) SmTi_3_(Sb,Sn)_4_, respectively, with several bands highlighted in white as a guide to the eye. Using the band crossing (black dashed line) as a reference, we derive a doping efficiency of 250 meV per (Sb,Sn) substitution. (c, d) Electronic band structure for hypothetical SmTi_3_Sn_4_ and SmTi_3_Sb_4_, showing several values of *E*_F_: The native stannide *E*_F_ (purple), the native antimonide *E*_F_ (red), and a gradient representing the experimental (Sb,Sn) alloy compositions (green Sb-rich, blue Sn-rich). (e, f) Density of states (D­(E)) and Crystal Orbital Hamilton Population (COHP) calculations for SmTi_3_Sn_4_ and SmTi_3_Sb_4_, demonstrating that alloys are stabilized by balancing energetic contributions from filling bonding states, depopulating antibonding states, and minimizing D­(*E*_F_). (g, h) Ti–Ti, Sn–Sn, and Sb–Sb interactions dominate the integrated COHP (ICOHP) for both compounds.

We can also approach the Fermi-level shift independently by calculating the expected shift in *E*_F_ for Sb-substituted SmTi_3_Sn_4_ and Sn-substituted SmTi_3_Sb_4_. While SmTi_3_Sn_4_ and SmTi_3_Sb_4_ do not exist, examining the hypothetical parent structures can provide significant insight. Figure 2(c,d) shows the DFT calculations for SmTi_3_Sn_4_ and SmTi_3_Sb_4_ over the pseudohexagonal Brillouin zone (for the pseudohexagonal zone projection, see refs 31,38 ). For each simulation, we show three key values of *E*_F_: (1) The native *E*_F_ for the pure stannide (purple), (2) the native *E*_F_ for the pure antimonide (red), and (3) a gradient of potential *E*_F_ given the (Sb,Sn) alloying (green Sb-rich, blue Sn-rich). To approximate the experimental doping window, we used the compositions Sn-rich SmTi_3_Sb_3.1_Sn_0.9_ and Sb-rich SmTi_3_Sb_3.9_Sn_0.1_. We calculate a doping window of 210 meV, corresponding to 263 meV per (Sb,Sn) substitution, in excellent agreement with the ARPES results.

The DFT results in Figure 2(c,d) also allow us to examine the general influence of (Sb,Sn) alloying on the bulk band structure. Qualitatively, the band structures of SmTi_3_Sn_4_ and SmTi_3_Sb_4_ are remarkably similar near the relevant *E*_F_, despite full substitution of Sb and Sn. This suggests that the (Sb,Sn) swapping likely has a minimal impact on the local electronic structure, particularly near *E*_F_. As additional support for the benign effect of (Sb,Sn) doping, we have included a series of additional ARPES measurements in the Supporting Information (Figure S1). Summarizing the results of the supplementary data, we have observed that constant-energy-contour plots (CECs) generated at *E*_F_ = 0 meV in the Sn-rich SmTi_3_(Sb,Sn)_4_ are very similar to the CECs generated at *E*_F_ = – 130 meV in the Sb-rich compound. This is most obvious around the Γ pocket, and suggests that the alloying primarily acts to tune *E*_F_.

Figure 2(e) presents D­(E) and COHP­(E) calculations for SmTi_3_Sn_4_. This analysis focuses on the Ti–Ti (two symmetry-unique) and Sn–Sn (nine symmetry-unique, summed) interactions as they contribute over 75% of the total integrated COHP (see Figure 2(g)). For completeness, all other interactions are provided in the Supporting Information (Figure S9). From D­(E), we can see that the native Fermi level (purple) is near a local maximum in D­(E). Electron doping via Sb substitution (purple to green/blue) adjusts *E*_F_ up and into a local minimum. Furthermore, electron doping populates additional Ti–Ti and Sn–Sn bonding states (shaded gray), further stabilizing the structure. The system reaches a natural limit in the electron-doped regime as further doping would increase D­(*E*_F_) and incorporate substantial antibonding contributions.

Figure 2(f) examines the DOS and COHP simulations for SmTi_3_Sb_4_. Again, we focus on the Ti–Ti and Sb–Sb interactions as they are the dominant contributions to the integrated COHP (Figure 2(h)). Unlike the stannide, the antimonide is already near a local minimum in D­(E). However, both the Ti–Ti and Sb–Sb interactions have substantial antibonding character at *E*_F_. The antimonide therefore experiences competition between maintaining low D­(*E*_F_) and the depopulation of antibonding states by lowering *E*_F_. This supports the experimentally observed alloying range (green/blue), which sits slightly below the natural *E*_F_ (red) for SmTi_3_Sb_4_. Despite the energetic tendency to continue depopulating antibonding states, further Sn doping is likely limited due to a rapidly increasing D­(*E*_F_).

From our analysis, we believe that the charge degree of freedom offered by the synergistic doping of (Sb,Sn) drives the stabilization of the *Ln*Ti_3_(Sb,Sn)_4_ series and explains the difficulty in realizing SmTi_3_Sb_4_ and SmTi_3_Sn_4_. Fortuitously, the (Sb,Sn) doping also seems to have a benign effect on the electronic structure near *E*_F_, providing a means to not only stabilize a new compound but also tune the Fermi level. As such, we will now examine the effects of Fermi-level tuning on the physical properties of the SmTi_3_(Sb,Sn)_4_ series.

### Properties of the SmTi_3_(Sb,Sn)_4_ Solid Solution

Kagome materials can exhibit many exotic electronic and magnetic phases, but these phases generally only emerge when the Fermi level is tuned correctly.^1–3,69^ Synergistic doping provides a potential route to both stabilize new crystal structures and control electron filling. Given the strong magnetic character of the *AM*_3_ *X*_4_ family, the (Sb,Sn) flexibility may provide a convenient means to tune the magnetic and electronic properties. In this section, we will investigate the effect of (Sb,Sn) synergistic doping on the magnetic properties of SmTi_3_(Sb,Sn)_4_. We will begin with a detailed investigation of the physical properties of Sb-rich (WDS: SmTi_3_Sb_3.86_Sn_0.14_) and Sn-rich (WDS: SmTi_3_Sb_3.33_Sn_0.67_) crystals. We will then briefly explore the compositional dependence across the alloy series on the magnetic ground state, deriving a basic magnetic phase diagram as a function of (Sb,Sn) alloying. Our ultimate goal is to demonstrate the potential value in developing phases with synergistic dopants, particularly those with complex magnetic and electronic properties.

Our discussion here focuses on the SmTi_3_(Sb,Sn)_4_ series of materials. However, many of the other members of the *Ln*Ti_3_(Sb,Sn)_4_ family also exhibit significant (Sb,Sn) substitution ranges, though the effect of alloying varies with rare-earth. For example, NdTi_3_(Sb,Sn)_4_ exhibits a tunable magnetic ground state while GdTi_3_(Sb,Sn)_4_ is less affected by the (Sb,Sn) content. Given the recent interest in the analogous bismuthides like GdTi_3_Bi_4_,^37,43,48,50^ and CeTi_3_Bi_4_,^34,38^ we have provided some basic magnetic characterization of the Nd, Gd, Pr, and Ce-based compounds in the Supporting Information (Figures S5–S8). A brief discussion of each can be found in the supplementary captions, which we hope can help guide further research into the *Ln*Ti_3_(Sb,Sn)_4_ family.

Figure 3 presents a summary of the magnetic, electronic, and thermodynamic properties for the Sn-rich and Sb-rich end points of the SmTi_3_(Sb,Sn)_4_ series. We begin our discussion by focusing on the Sb-rich compound (green). Figure 3(a) demonstrates the field-cooled (FC) and zero-field-cooled (ZFC) magnetization curves for a single crystal with the field oriented parallel to the three principal axes. SmTi_3_(Sb,Sn)_4_ exhibits a highly anisotropic magnetic response with a clear easy *c*-axis magnetization. Qualitatively, the magnetization resembles a ferromagnetic (FM) system with a primary transition around 10 K and clear FC/ZFC splitting. While the ZFC measurements show additional features below 10 K, these are likely related to magnetic domain formation. FC measurements will be used when referring to all magnetic transition temperatures. A weak shoulder (approximately 11 K, see inset) is observed immediately before the main transition.

**Figure 3:**
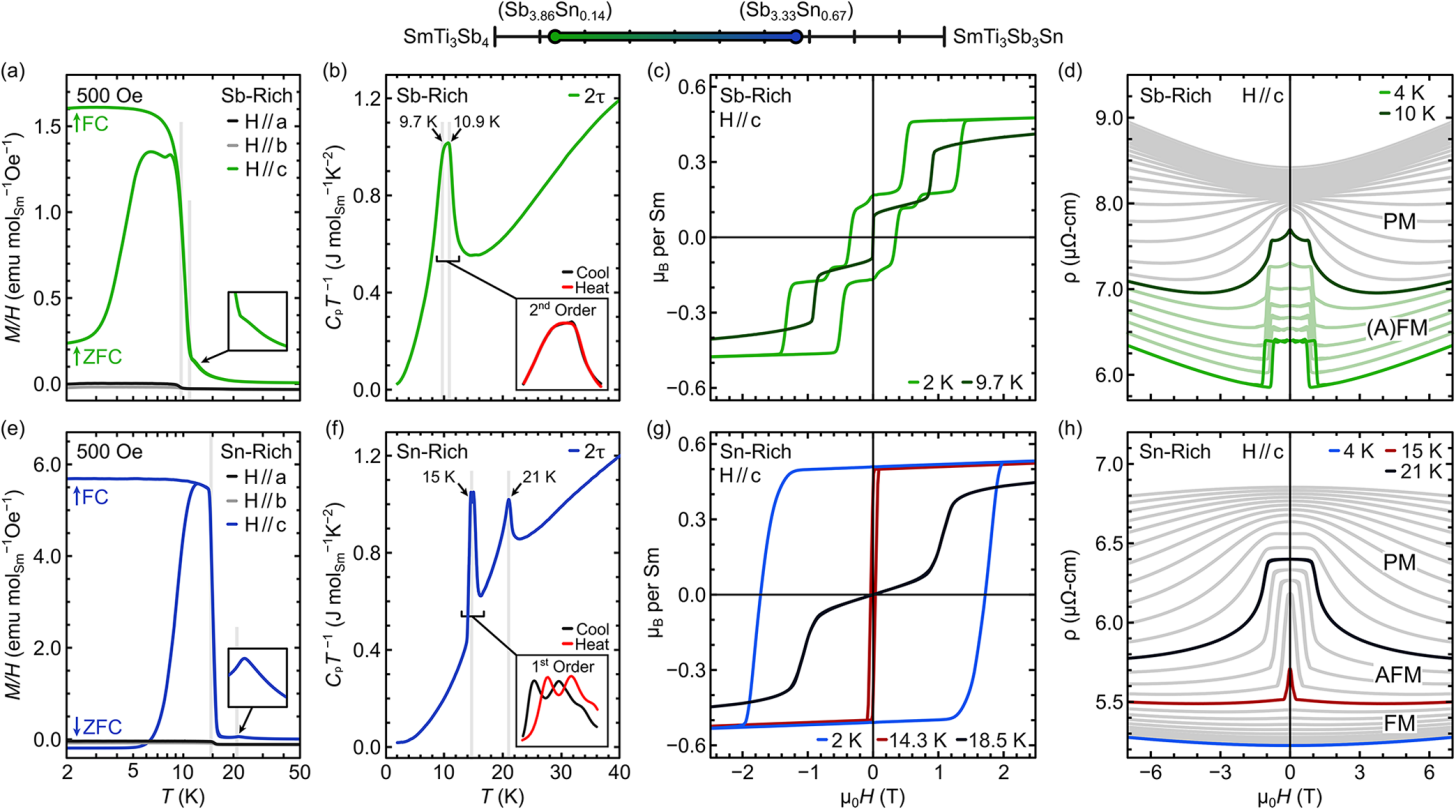
Fundamental properties of the Sb-rich (green) and Sn-rich (blue) termini of the SmTi_3_(Sb,Sn)_4_ solid solution. (a, e) Field-cooled and zero-field-cooled magnetization along three principal crystallographic directions, highlighting a cusp followed by a steep plateau in both samples. (b, f) Large-pulse zero-field heat capacity measurements. Both samples show two transitions, marked by gray lines and mirrored in the magnetization plots. Insets show heating and cooling branches of the single-slope heat capacity analysis, demonstrating that the Sn-rich 15 K peak is first-order. (c, g) Isothermal magnetization results with H∥c. At least one intermediate temperature is shown to highlight the vanishing of the FM-like hysteretic component. (d, h) Magnetotransport measurements showing the emergence of negative magnetoresistance (negative MR) from 4 to 30 K in the AFM state for both samples. Negative MR is suppressed entirely when the Sn-rich sample transitions to the FM state.

Heat capacity measurements reveal a broad peak coinciding with the magnetization onset around 10 K. Closer analysis of the heat capacity peak indicates that it is a convolution of two peaks separated by approximately 1 K. Lack of splitting in the heating and cooling branches of the high-pulse single-curve slope analysis indicates that both peaks are second order (see inset). The centroids of these peaks correspond to the weak shoulder and primary transition temperature in the magnetization results. We have highlighted the peak temperatures in both the magnetization and heat capacity results with light gray bars fo cross-reference.We were unable to make either the La- or Ca-based analogs, so our estimate of the magnetic entropy utilizes a simple third-order polynomial to approximate the nonmagnetic heat capacity signal. We fit the polynomial to the range of 1.8–4 and 22–40K, yielding an approximate entropy release of 75-80% of Rln(2), consistent with prior results for SmTi_3_Bi_4_.^31^ Plots of the approximated magnetic heat capacity and magnetic entropy are included in the Supporting Information (Figure S10).

Heat capacity and magnetization results suggest two closely interacting magnetic transitions. To investigate, we performed isothermal magnetization measurements at (1) the base temperature of 2 K, and (2) an intermediate temperature of 9.7 K (at the onset of FC/ZFC splitting). By 2 K, the system exhibits a blend of AFM and FM states, mixing multiple metamagnetic plateaus with clear coercivity. At 9.7 K (and above), the coercivity vanishes, and the system resembles a more prototypical AFM material. Thus, upon cooling the system first transitions into an AFM-dominated state below 10.9 K and then eventually develops FM-like correlations which impart substantial coercivity below 9.7 K. These effects can also be observed in the symmetrized, transverse magnetoresistance (Figure 3(d)), where we can observe the sharp onset of negative MR (dark green curve) with a critical field that matches the 0.9 T spin flop transition. Hysteresis develops continuously upon cooling (green curves) below 9.7 K. To aid graphical visibility, the light green curves below 10 K have been manually offset from each other by a slight *y*-axis offset (the original version can be found in Figure S10).

We now turn to the Sn-rich (blue) end point. As established in the ARPES and DFT data, these compositions differ by approximately 0.5 Sn (0.5 holes) per formula unit, with only a very weak (0.3%) response in the crystallographic parameters and minimal changes to the electronic structure. We anticipate that modifications to the magnetic ground state are primarily induced by changes in the electronic filling due to synergistic doping. Figure 3(e) presents the FC/ZFC magnetization results, demonstrating a highly anisotropic FM-like response with easy *c*-axis magnetization at 15 K. A clear, albeit weak, cusp is visible at 21 K (inset).

Heat capacity measurements (Figure 3(f)) show two distinct thermodynamic signatures corresponding to the FM-like rise and AFM-like cusp (marked by gray lines on Figure 3(e,f)). Splitting of the heating and cooling high-pulse single-curve slope analysis (inset) demonstrates that the 15 K peak is first-order. Figure 3(g) shows isothermal magnetization measurements performed at 18.5 K (black), confirming the onset of AFM order below 21 K. At 14.3 K (red), immediately below the 15 K transition, the AFM order appears to be completely suppressed and replaced with FM-like behavior. The system’s coercivity continues to increase down to 2 K, where the coercive field is approximately 2 T.

We note that the saturation magnetization is not modified substantially between the Sb-rich and Sn-rich compositions, suggesting that the (Sb,Sn) is modifying the magnetic interactions, but not the effective moment or crystalline electric fields (CEF) of the Sm ion. The saturation magnetization in both systems (and throughout the alloy series, as we will shortly see) remains around 0.5 μ_B_ per Sm, which is less than the expected free moment (*gJ* = 0.71 μ_B_). Our previous measurements on SmTi_3_Bi_4_ show a similar deviation from the expected free-ion moment.^31^ Deviations from *gJ* are common in Sm-containing intermetallics due to the sensitivity of the Sm^3+^ ion to crystal field effects.^70^

Unlike the hybridized state in Sb-rich SmTi_3_(Sb,Sn)_4_, magnetic interactions in the Sn-rich composition are more indicative of competition between the intermediate AFM- and FM-like ground states. This is evident in the electronic transport data (Figure 3(h)). Both the Sb-rich (green) and Sn-rich (blue) samples develop negative MR as we cool from the paramagnetic state to the intermediate AFM state. In the Sn-rich system, however, the negative MR response is quickly suppressed at below 15 K. These results are consistent with the first-order nature of the 15 K heat capacity peak, provide strong evidence for the competition between AFM and FM states, and confirm that there is no symmetry-allowed continuous route for this transition.

As supporting evidence for the fundamental difference between the Sb-rich and Sn-rich SmTi_3_(Sb,Sn)_4_ compositions, we collected a series of field-dependent heat capacity and temperature-dependent magnetization measurements on both samples. We have elected to provide these results in the Supporting Information (Figure S11), as they are graphically quite complex and do not reveal any new transitions that cannot be inferred from Figure 3. However, the field-dependent data clearly demonstrates that the Sb-rich composition exhibits two peaks with different field dependencies. Furthermore, the field evolution helps clarify the nature of the heat capacity peaks in the Sn-rich composition. The proposed AFM peak is suppressed with field while the FM-like peak is enhanced. The peaks merge shortly before transitioning to a high-field (polarized) state. Much of this data can be visualized in two preliminary temperature-field phase diagrams presented in the Supporting Information (Figure S11­(c,f)), which we hope can help guide future research efforts.

Before continuing to a broader composition-dependent comparison Figure 4, we will briefly summarize the properties of the Sb-rich (green) and Sn-rich (blue) end points. The Sn-rich (blue) composition exhibits competition between AFM and FM order. The AFM order emerges first but is ultimately suppressed and replaced by the FM order. The Sb-rich (green) composition experiences the emergence of AFM order with multiple metamagnetic steps around 11 K and then continually adds coercivity to the AFM state, creating a hybrid ground state. We suspect that the ground state of the system is likely either (1) AFM with substantial spin canting, (2) a ferrimagnet, or (3) an AFM with evolution of a spin-density wave. At present, we have insufficient data to definitively characterize the magnetic nature of the Sb-rich SmTi_3_(Sb,Sn)_4_. For brevity, we will refer to this state as A­(FM). Unfortunately, Sm is a very poor isotope for neutron diffraction studies, so we believe that the application of microscopic techniques may be invaluable for determining magnetic interactions in the SmTi_3_(Sb,Sn)_4_ family.

Thus far, we have only shown the Sb-rich and Sn-rich termini of the SmTi_3_(Sb,Sn)_4_ solid solution. However, since the (Sb,Sn) alloying is a continuous process, we were curious whether we can determine a “crossover” composition at which the system switches between the Sb-rich and Sn-rich behavior. The ability to tune and control the compositions of these systems is one of the key benefits of intermetallics developed using the synergistic doping methodology.

Figure 4 shows the physical properties of a series of SmTi_3_(Sb,Sn)_4_ single crystals ranging between the Sb-rich and Sn-rich termini in roughly equivalent (Sb,Sn) steps. From left to right, we display (1) sample names and explicit (Sb,Sn) compositions as determined by EDS, (2) temperature-dependent magnetization superimposed with zero-field heat capacity, and (3) isothermal magnetization curves at base temperature (color) and an intermediate temperature (gray). For graphical simplicity, all results are shown in arbitrarily scaled units, though a corresponding set of quantitative plots is provided in the Supporting Information (Figure S4).

**Figure 4:**
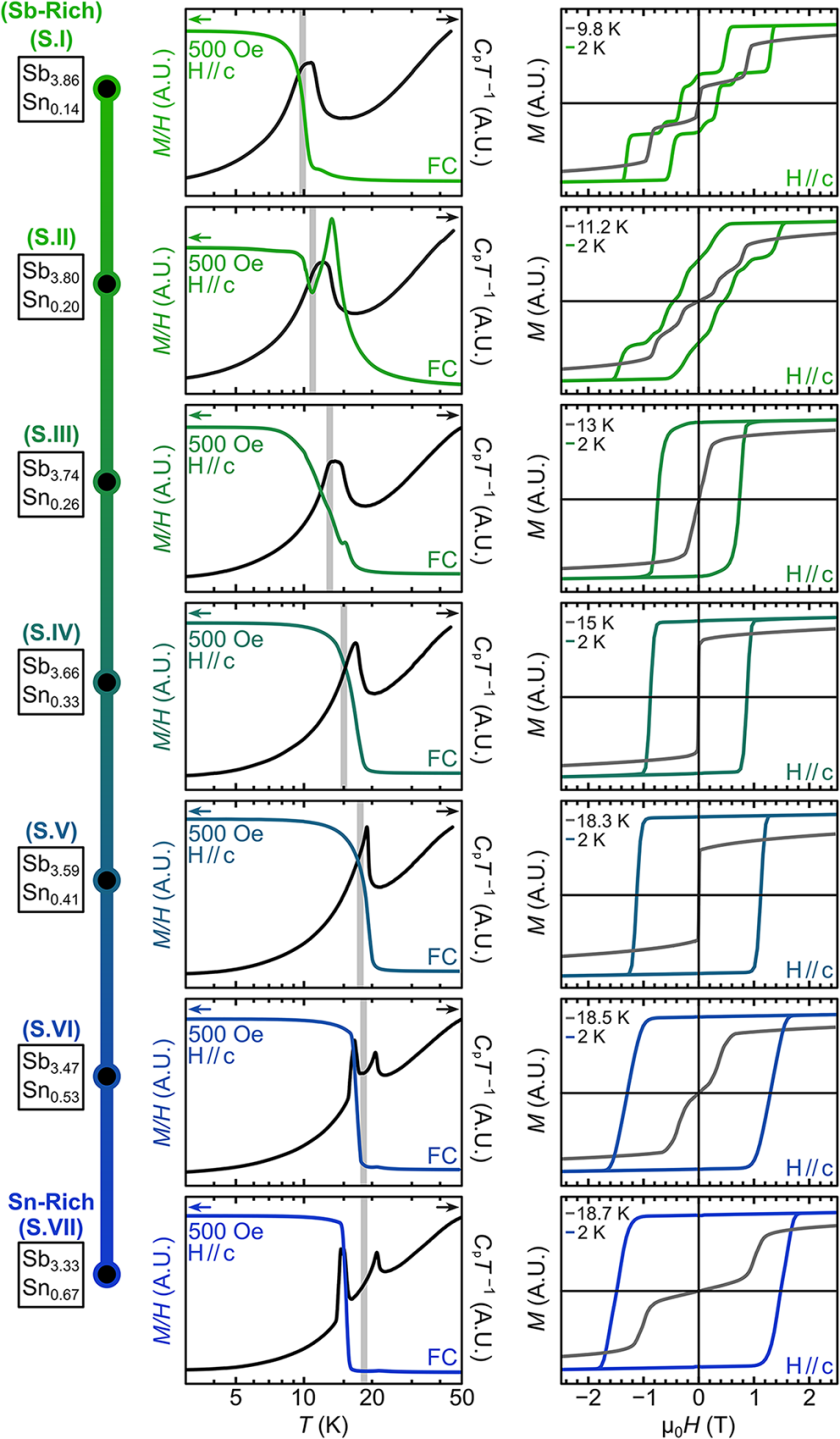
Summary of samples traversing the SmTi_3_(Sb,Sn)_4_ solid solution to demonstrate the gradual shift in magnetic and thermodynamic properties. Shown from left to right are (1) the measured (Sb,Sn) content of the single crystals, (2) temperature-dependent magnetization and zero-field heat capacity results superimposed and scaled in arbitrary units, and (3) isothermal magnetization curves at base temperature and an intermediate temperature (gray) intended to demarcate the transition between the two heat capacity features (where possible). For clarity, the intermediate temperature (gray) is also marked on the temperature-dependent magnetization.

As discussed above, the Sb-rich sample (S.I., 3.5% Sn) hosts the A­(FM) state, which blends the metamagnetic plateaus from the 10.9 K AFM transition and substantial coercivity from the subsequent 9.7 K transition. Here, we have well-defined metamagnetic plateaus in both the base and intermediate temperatures alongside a broad heat capacity peak composed of two individual contributions. The characteristic broadness of the heat capacity peak is maintained at sample S.II (5.0% Sn), though the sharpness of the metamagnetic transitions degrades. The number of plateaus also changes in the intermediate AFM state, from two well-defined plateaus at S.I to approximately three plateaus in S.II. By sample S.III (6.5% Sn), the metamagnetic plateaus have effectively vanished. The 2 K isothermal magnetization is replaced by a coercive FM loop with distinct rounded corners. Despite sweeping through a range of temperatures, we were unable to find any temperature at which the isothermal magnetization exhibited the metamagnetic plateaus characteristic of the intermediate-temperature regime seen in both the Sb-rich and Sn-rich examples from Figure 3.

For all compositions past this point, increasing concentrations of Sn appear to increase the coercivity of the isothermal magnetization loop at 2 K. However, subtle changes are evident in the heat capacity and intermediate (gray) isothermal magnetization loops. At S.IV (8.2% Sn), both the heat capacity peak and the isothermal magnetization curve began to sharpen. By sample S.V. (10.2% Sn), it is difficult to resolve the split heat capacity peak, and the magnetization at 2 K looks very similar to a prototypical ferromagnet. Upon reaching sample S.VI (13.3% Sn), the heat capacity peak clearly breaks into two peaks (the higher-temperature AFM transition and the lower-temperature first-order FM-like transition). Concomitant with the heat capacity splitting, we recovered the characteristic metamagnetic plateaus at an intermediate temperature of 18.5 K. The final composition S.VII (16.8% Sn) is the same sample as the Sn-rich SmTi_3_(Sb,Sn)_4_ shown in Figure 3 and exhibits clear competition between the intermediate AFM state and the FM-like ground state.

These results highlight the complex relationship between the (Sb,Sn) synergistic doping and the magnetic ground state in the SmTi_3_(Sb,Sn)_4_ series. Figure 5 summarizes the results of Figure 4 (and several additional intermediate compositions) as a composition-dependent magnetic phase diagram. Results derived from both heat capacity measurements (Figure 5(a)) and temperature-dependent magnetization measurements (Figure 5(b)) are shown. Heat capacity measurements resolve the subtle peak splitting and can also distinguish between first- and second-order transitions. The inset helps indicate the color scheme for the samples. For samples toward the Sn-rich end member, blue points indicate the higher-temperature AFM peak and green points indicate the lower-temperature (first-order) FM peak. As demonstrated in Figures 3(a, inset) and 4, Sb-rich compositions always exhibit a broad heat capacity peak weakly split into two contributions. These two temperatures are indicated by the red points. Gray lines are added as guides to the eye.

**Figure 5:**
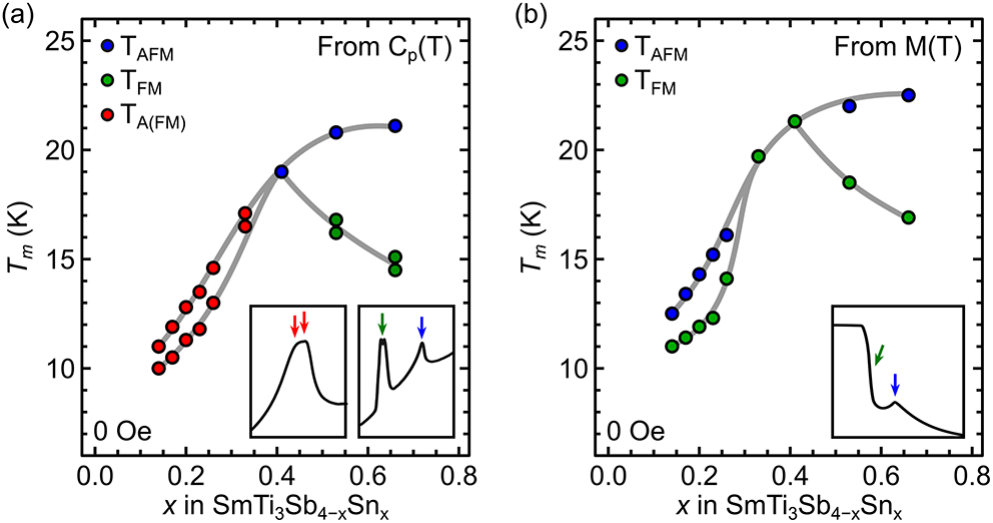
Summary of the temperature–composition phase diagrams for the SmTi_3_(Sb,Sn)_4_ solid solution. Diagrams are derived from (a) heat capacity and (b) magnetization measurements. Both plots demonstrate the competition between the higher-temperature AFM order and the onset of FM order at lower temperatures.

The phase diagram produced via magnetization results is slightly harder to interpret because the strength of the FM transition often obscures the intermediate AFM cusp. Unfortunately, due to the myriad of other features that also emerge in the intermediate composition regime (see sample S.II and S.III in Figure 4), the temperature derivative of the FC data is also unreliable. We caution that it is easy to overanalyze the magnetization data, particularly with the numerous subtle and weak features. For example, consider that measurements made in Figure 4 were performed at 500 Oe. For some of the samples (e.g., S.IV), a field of 500 Oe will induce full saturation of the moment at 15 K, and for some samples (e.g., S.VII), the field will be in a decidedly unsaturated regime. Further, with the known presence of many metamagnetic plateaus, it is challenging to analyze subtle features in each composition without full field-temperature phase maps. As such, we are very conservative when estimating phase transition temperatures in the magnetization data. We restrict our analysis to the two features, which are present in almost all temperature-dependent magnetization measurements: (1) A small, weak cusp at higher temperatures, and (2) a much stronger FM-like transition and plateau. If we define *T*_AFM_ as the cusp peak and *T*_FM_ as the midpoint of the FM transition, then we can generate the magnetization-derived phase diagram shown in Figure 5(b).

While our composition-dependent phase diagrams are qualitatively interesting results, the full solution of the SmTi_3_(Sb,Sn)_4_ phase diagram was not the primary goal of this paper. Instead, our goal was to demonstrate the potential value of synergistic doping as a technique to develop new complex intermetallics while simultaneously tuning their electronic and magnetic properties. Our results have highlighted how the intrinsic tunability inherited from the (Sb,Sn) solid solution imparts a complex and nuanced degree of freedom to the SmTi_3_(Sb,Sn)_4_ family of kagome metals. Not only does the (Sb,Sn) synergistic doping stabilize the crystal structure through controlling the electron filling and the relevance of bonding/antibonding states, but we also gain the ability to tune and control the interplay between the AFM and FM interactions intrinsic to the system.

## Conclusions

In this work, we presented the single-crystal synthesis and characterization of the *Ln*Ti_3_(Sb,Sn)_4_ series, a family that forms only as a solid solution between the hypothetical *Ln*Ti_3_Sn_4_ and *Ln*Ti_3_Sb_4_ end points. We demonstrated controllable incorporation of (Sb,Sn) into the single crystals, and characterized the solubility range across the rare-earth series (*Ln*: Ce–Gd). We proposed the idea of “synergistic doping” to explain the stabilizing effect of the (Sb,Sn) alloy, and used a combination of ARPES, DFT, and COHP measurements to test the concept. We found that the synergistic doping of (Sb,Sn) provides a natural way to control *E*_F_, and allows the *Ln*Ti_3_(Sb,Sn)_4_ system to balance the minimization of D­(*E*_F_) with the occupation of bonding and antibonding states. The tunable *E*_F_ also had a profound effect on the electronic and magnetic properties, which we explored through a detailed characterization of the SmTi_3_(Sb,Sn)_4_ series. We demonstrated a complex, tunable interaction between competing AFM and FM ground states. While the precise nature of these magnetic states is still unknown, they exemplify the potential value of designing new intermetallics using synergistic doping.

Ultimately, this work accomplishes two goals: (1) It presents a new family of cleavable, air-stable kagome metals with complex magnetic ground states and strong potential for correlated phenomena, and (2) it establishes the *Ln*Ti_3_(Sb,Sn)_4_ series as a model system for establishing synergistic doping as a synthetic strategy toward tunable, complex intermetallics.

## Supplementary Material



## Notes

ARPES data on the Sb-rich and Sn-rich end points; ARPES data showing comparison with SmTi_3_Bi_4_; decomposed density of states calculations; crystallography summary tables for uploaded CIF files; summary table and graphic for neutron scattering experiment; summary of rare-earth volumetric expansion and de Gennes scaling plot; supplementary characterization data for NdTi_3_(Sb,Sn)_4_, GdTi_3_(Sb,Sn)_4_, CeTi_3_(Sb,Sn)_4_, and PrTi_3_(Sb,Sn)_4_; full set of COHP pairwise interactions; magnetic heat capacity and entropy approximation for Sm-rich SmTi_3_(Sb,Sn)_4_; magnetic-field-dependent heat capacity and magnetization for Sb-rich and Sn-rich SmTi_3_(Sb,Sn)_4_; full set of MvH, MvT, CvT curves for alloy series with quantitative axes; and precession images for Sn-rich SmTi_3_(Sb,Sn)_4_ (PDF)
