# Supplementary material for: Synergistic Doping and Stabilization of Magnetically Tunable LnTi3(Sb,Sn)4 (Ln: Ce–Gd) Kagome Metals
Source: J Am Chem Soc. 2026 Jul 23;148(30):32014–25. doi: 10.1021/jacs.6c06163 (PMC13450405; doi:10.1021/jacs.6c06163)
Supplement: Supplementary file 1 [file ja6c06163_si_001.pdf]

Supplementary information for: Synergistic doping and stabilization of magnetically tunable LnTi$_3$(Sb,Sn)$_4$ (Ln:Ce–Gd) kagome metals

Brenden R. Ortiz,[1,*] Ramakanta Chapai,[1,2] German Samolyuk,[1] Milo Sprague,[3] Arun K. Kumay,[3] Hu Miao,[1] Karolina Górnicka,[1,4] Xiaoping Wang,[5] Qiang Zhang,[5] Madhab Neupane,[3] David Parker,[1] and Jiaqiang Yan[1]

[1]*Materials Science and Technology Division, Oak Ridge National Laboratory, Oak Ridge, TN 37831, USA*
[2]*Department of Physics, Norfolk State University, Norfolk, VA 23504, USA*
[3]*Department of Physics, University of Central Florida, Orlando, FL 32816, USA*
[4]*Applied Physics and Mathematics Department, Advanced Materials Centre, Gdansk University of Technology, ul. Narutowicza 11/12, 80-233 Gdansk, Poland*
[5]*Neutron Scattering Division, Oak Ridge National Laboratory, Oak Ridge, TN 37831, USA*

(Dated: July 10, 2026)

Here we present our synthesis and characterization of the LnTi$_3$(Sb,Sn)$_4$ (Ln: Ce, Pr, Nd, Sm, Gd) family of cleavable kagome metals. While these materials are isostructural to the LnTi$_3$Bi$_4$ family, they only form as (Sb,Sn) solid solutions with no corresponding LnTi$_3$Sb$_4$ or LnTi$_3$Sn$_4$ phases. We use a combination of first-principles density functional theory (DFT) and crystal orbital Hamilton population (COHP) calculations to show that (Sb,Sn) alloying has a stabilizing effect on the structure by adjusting the Fermi level, filling bonding states, depopulating antibonding states, and adjusting the density of states (DOS) towards local minima, an effect we call "synergistic doping." Through a detailed characterization of the SmTi$_3$(Sb,Sn)$_4$ series, we further demonstrate that the tunable Fermi level has a profound effect on the physical properties. We observe multiple magnetic phases which stem from a competition between antiferromagnetic (AFM) and ferromagnetic-like (FM) ground states. Furthermore, the (Sb,Sn) ratio allows us to tune from direct AFM-FM competition towards a complex admixture with properties reminiscent of the recently discovered TbTi$_3$Bi$_4$. Ultimately, our work demonstrates how the concept of synergistic doping provides a means to stabilize new structures while developing systems with intrinsically tunable chemical, magnetic, and electronic properties.

* ortizbr@ornl.gov

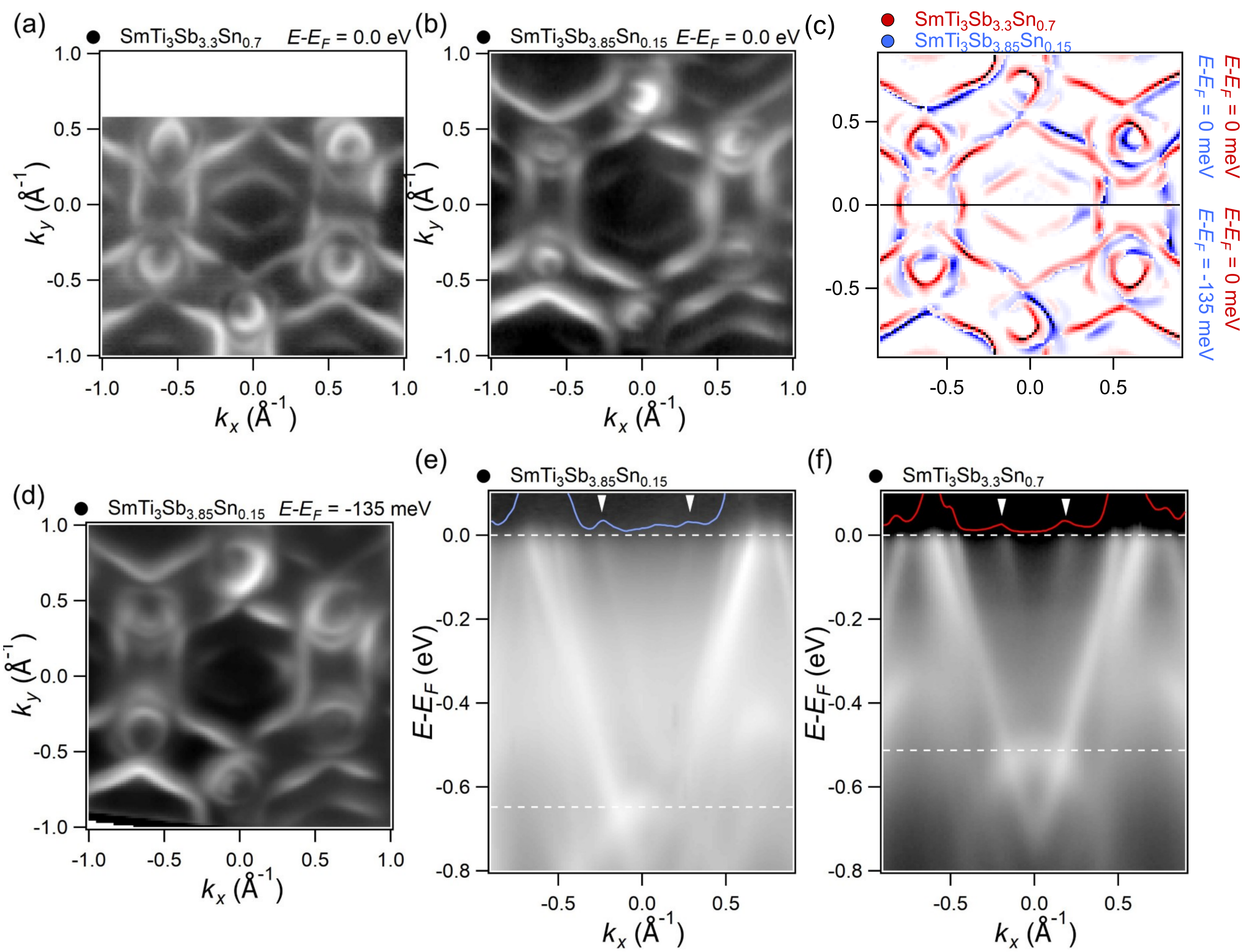

FIG. S1. Supplementary ARPES data collected on the Sn-Rich and Sb-rich $SmTi_3(Sb,Sn)_4$ series. (a) Constant energy contour (CEC) for Sb-rich $SmTi_3(Sb,Sn)_4$ at the natural Fermi level. (b) CEC for Sn-rich $SmTi_3(Sb,Sn)_4$ at the natural Fermi level. While the Sb-rich and Sn-rich compounds look broadly similar, there is a clear shift in the bands near Γ. (c) Comparison between the Sn-rich and Sb-rich ARPES scans at the natural Fermi level (top) and the same materials but with the Sb-rich CEC shifted to the -135 meV level. We can see that the bands near Γ are much more aligned with the 135 meV shift. This shift corresponds to the doping difference between the Sb-rich and Sn-rich $SmTi_3(Sb,Sn)_4$. (d) CEC of the Sb-rich compound at the -135 meV Fermi level. (e,f) Same ARPES data shown in the main body, highlighting the bands near Γ and the Fermi level shift induced by the Sb-Sn doping.

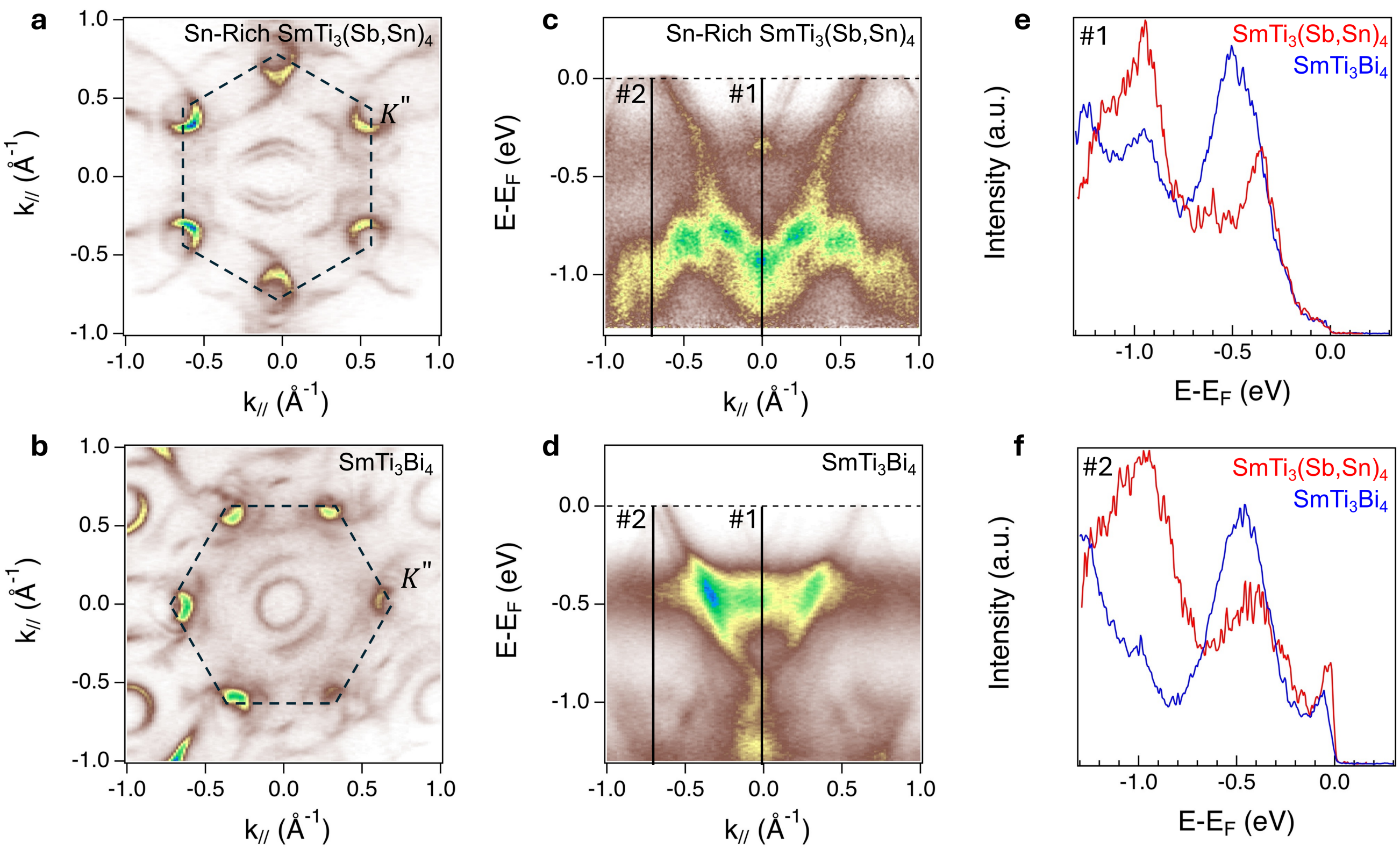

FIG. S2. Supplementary ARPES data collected on the Sb-rich $SmTi_3(Sb,Sn)_4$and the bismuthide $SmTi_3Bi_4$. (a,b) Constant energy contour (CEC) ARPES data for $SmTi_3(Sb,Sn)_4$ and $SmTi_3Bi_4$, respectively. Dashed hexagons highlight the Brillouin zone boundary on the surface. (c,d) ARPES intensity plots along K-Γ-K direction, corresponding to the red dashed line shown in CEC plots. (e) Comparison of the energy distribution curves (EDCs) at the G point (cut 1). Blue and red EDCs represent $SmTi_3Bi_4$ and $SmTi_3(Sb,Sn)_4$, respectively. (f) Comparison of EDCs at the K point (cut 2). Comparing with $SmTi_3Bi_4$, the shift in the electronic structure of $SmTi_3(Sb,Sn)_4$ is consistent with hole doping in $SmTi_3(Sb,Sn)_4$.

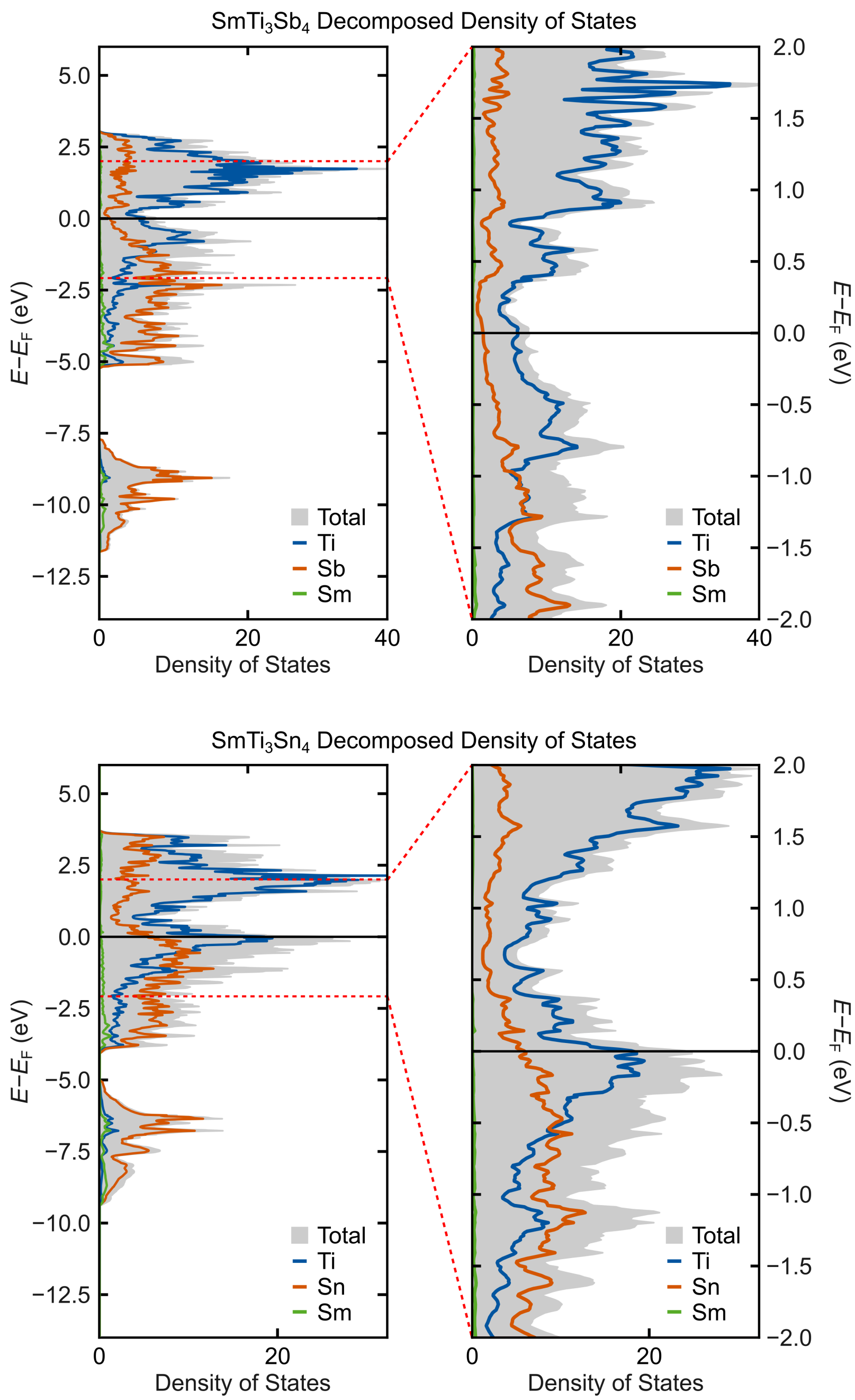

FIG. S3. Supplementary atom decomposed density of states used to examine the relative location of the Sm orbitals. Since the Sm orbital contribution is fairly deep below E_F, we believe that the nonmagnetic simulations will provide a relatively accurate assessment of the band structure near E_F.

TABLE S1. Abbreviated summary of supplementary crystal data, structural refinement, and atomic positions for Sb-rich $SmTi_3(Sb,Sn)_4$. Specific composition is $Sm_8Ti_{24}Sb_{30.87}Sn_{1.13}$ (corresponding to green data traces in main body). Complete CIF file is provided in the supplementary information. Specific (Sb,Sn) composition was set using results from energy dispersive spectroscopy. Preliminary neutron diffraction on $NdTi_3(Sb,Sn)_4$ suggests that there is no preferential occupation of Sn and no structural superlattice from potential (Sb,Sn) ordering. As such, we choose to provide CIF files as simple, homogenous alloys where (Sb,Sn) is shared among all anion sites.

Crystal Data and Refinement		Atomic Positions (fractional coordinates)					
Property	Value	Label	x	y	z	U_{iso}	Occ.
Formula	$\mathbf{Sm_8Ti_{24}Sb_{30.87}Sn_{1.13}}$	Sm01	1	1/2	0.69542(2)	0.00787(9)	1
Space group, Z	Fmmm, 1	Ti01	1/2	1/2	0.59182(4)	0.0069(2)	1
a (Å)	5.7619(11)	Ti02	1/4	1/4	0.40441(3)	0.00645(16)	1
b (Å)	10.0832(18)	Sb01	1/2	0.32899(4)	1/2	0.00759(10)	0.9647
c (Å)	24.047(4)	Sn01	”	”	”	”	0.0353
Volume (Å^3)	1397.1(4)	Sb02	0	0.15938(3)	0.31150(2)	0.00743(9)	0.9647
Density (calc.) (g cm^{-3})	7.422	Sn02	”	”	”	”	0.0353
R_{int}, Completeness	0.0173, 99.7%	Sb03	1	1/2	0.56645(2)	0.00722(10)	0.9647
R_1 $[I > 2\sigma(I)]$	0.0162	Sn03	”	”	”	”	0.0353
wR_2 $[I > 2\sigma(I)]$	0.0420						
Largest peak/hole (e Å^{-3})	1.217 / -1.454						
GOF / WGHT (A,B)	1.08 / (0, 5.5)						

TABLE S2. Abbreviated summary of supplementary crystal data, structural refinement, and atomic positions for Sn-rich $SmTi_3(Sb,Sn)_4$. Specific composition is $Sm_8Ti_{24}Sb_{26.70}Sn_{5.30}$ (corresponding to blue data traces in main body). Complete CIF file is provided in the supplementary information. Specific (Sb,Sn) composition was set using results from energy dispersive spectroscopy. Preliminary neutron diffraction on $NdTi_3(Sb,Sn)_4$ suggests that there is no preferential occupation of Sn and no structural superlattice from potential (Sb,Sn) ordering. As such, we choose to provide CIF files as simple, homogenous alloys where (Sb,Sn) is shared among all anion sites.

Crystal Data and Refinement		Atomic Positions (fractional coordinates)					
Property	Value	Label	x	y	z	U_{iso}	Occ.
Formula	$\mathbf{Sm_8Ti_{24}Sb_{26.70}Sn_{5.30}}$	Sm01	1	1/2	0.69511(2)	0.00690(5)	1
Space group, Z	Fmmm, 1	Ti01	1/2	1/2	0.59265(2)	0.00576(8)	1
a (Å)	5.75920(10)	Ti02	1/4	1/4	0.40368(2)	0.00584(7)	1
b (Å)	10.0698(2)	Sb01	1/2	0.32901(2)	1/2	0.00796(5)	0.8345
c (Å)	24.1462(4)	Sn01	”	”	”	”	0.1655
Volume (Å^3)	1400.33(4)	Sb02	0	0.16003(2)	0.31104(2)	0.00651(5)	0.8345
Density (calc.) (g cm^{-3})	7.390	Sn02	”	”	”	”	0.1655
R_{int}, Completeness	0.0072, 99.2%	Sb03	1	1/2	0.56617(2)	0.00674(5)	0.8345
R_1 $[I > 2\sigma(I)]$	0.0072	Sn03	”	”	”	”	0.1655
wR_2 $[I > 2\sigma(I)]$	0.0204						
Largest peak/hole (e Å^{-3})	0.903 / -0.708						
GOF / WGHT (A,B)	1.17 / (0, 3.3)						

TABLE S3. Abbreviated summary of supplementary crystal data, structural refinement, and atomic positions for Sb-rich $CeTi_3(Sb,Sn)_4$. Specific composition is $Ce_8Ti_{24}Sb_{27.04}Sn_{4.96}$ (corresponding to green data traces in main body). Complete CIF file is provided in the supplementary information. Specific (Sb,Sn) composition was set using results from energy dispersive spectroscopy. Preliminary neutron diffraction on $NdTi_3(Sb,Sn)_4$ suggests that there is no preferential occupation of Sn and no structural superlattice from potential (Sb,Sn) ordering. As such, we choose to provide CIF files as simple, homogenous alloys where (Sb,Sn) is shared among all anion sites.

Crystal Data and Refinement		Atomic Positions (fractional coordinates)					
Property	Value	Label	x	y	z	U_{iso}	Occ.
Formula	$\mathbf{Ce_8Ti_{24}Sb_{27.04}Sn_{4.96}}$	Ce01	1	1/2	0.69519(2)	0.00915(17)	1
Space group, Z	Fmmm, 1	Ti01	1/2	1/2	0.59175(6)	0.0095(3)	1
a (Å)	5.8086(4)	Ti02	1/4	1/4	0.40521(4)	0.0089(3)	1
b (Å)	10.1072(5)	Sb01	1/2	0.32942(6)	1/2	0.01102(19)	0.845
c (Å)	24.3834(14)	Sn01	”	”	”	”	0.155
Volume (Å^3)	1431.52(15)	Sb02	0	0.16120(4)	0.31355(2)	0.00913(17)	0.845
Density (calc.) (g cm^{-3})	7.136	Sn02	”	”	”	”	0.155
R_{int}, Completeness	0.0215, 98.9%	Sb03	1	1/2	0.56515(2)	0.00983(19)	0.8455
R_1 $[I > 2\sigma(I)]$	0.0208	Sn03	”	”	”	”	0.155
wR_2 $[I > 2\sigma(I)]$	0.0650						
Largest peak/hole (e Å^{-3})	2.277 / -1.330						
GOF / WGHT (A,B)	1.15 / (0, 21.5)						

TABLE S4. Abbreviated summary of supplementary crystal data, structural refinement, and atomic positions for Sb-rich $PrTi_3(Sb,Sn)_4$. Specific composition is $Pr_8Ti_{24}Sb_{26.92}Sn_{5.08}$ (corresponding to green data traces in main body). Complete CIF file is provided in the supplementary information. Specific (Sb,Sn) composition was set using results from energy dispersive spectroscopy. Preliminary neutron diffraction on $NdTi_3(Sb,Sn)_4$ suggests that there is no preferential occupation of Sn and no structural superlattice from potential (Sb,Sn) ordering. As such, we choose to provide CIF files as simple, homogenous alloys where (Sb,Sn) is shared among all anion sites.

Crystal Data and Refinement		Atomic Positions (fractional coordinates)					
Property	Value	Label	x	y	z	U_{iso}	Occ.
Formula	$\mathbf{Pr_8Ti_{24}Sb_{26.92}Sn_{5.08}}$	Pr01	1	1/2	0.69505(2)	0.00799(9)	1
Space group, Z	Fmmm, 1	Ti01	1/2	1/2	0.59215(3)	0.00775(16)	1
a (Å)	5.7918(2)	Ti02	1/4	1/4	0.40455(2)	0.00733(12)	1
b (Å)	10.0937(4)	Sb01	1/2	0.32924(3)	1/2	0.00968(10)	0.8412
c (Å)	24.3230(9)	Sn01	”	”	”	”	0.1588
Volume (Å^3)	1421.94(9)	Sb02	0	0.16094(2)	0.31266(2)	0.00792(9)	0.8412
Density (calc.) (g cm^{-3})	7.191	Sn02	”	”	”	”	0.1587
R_{int}, Completeness	0.0145, 99.5%	Sb03	1	1/2	0.56528(2)	0.00823(9)	0.8412
R_1 $[I > 2\sigma(I)]$	0.0143	Sn03	”	”	”	”	0.1588
wR_2 $[I > 2\sigma(I)]$	0.0392						
Largest peak/hole (e Å^{-3})	1.962 / -1.027						
GOF / WGHT (A,B)	1.08 / (0, 8.4)						

TABLE S5. Abbreviated summary of supplementary crystal data, structural refinement, and atomic positions for Sb-rich $NdTi_3(Sb,Sn)_4$. Specific composition is $Nd_8Ti_{24}Sb_{26.32}Sn_{5.68}$ (corresponding to green data traces in main body). Complete CIF file is provided in the supplementary information. Specific (Sb,Sn) composition was set using results from energy dispersive spectroscopy. Preliminary neutron diffraction on $NdTi_3(Sb,Sn)_4$ suggests that there is no preferential occupation of Sn and no structural superlattice from potential (Sb,Sn) ordering. As such, we choose to provide CIF files as simple, homogenous alloys where (Sb,Sn) is shared among all anion sites.

Crystal Data and Refinement		Atomic Positions (fractional coordinates)					
Property	Value	Label	x	y	z	U_{iso}	Occ.
Formula	$\mathbf{Nd}_8\mathbf{Ti}_{24}\mathbf{Sb}_{26.32}\mathbf{Sn}_{5.68}$	Nd01	1	1/2	0.69510(2)	0.00771(9)	1
Space group, Z	Fmmm, 1	Ti01	1/2	1/2	0.59223(3)	0.00716(14)	1
a (Å)	5.7821(4)	Ti02	1/4	1/4	0.40437(2)	0.00683(12)	1
b (Å)	10.0828(6)	Sb01	1/2	0.32918(2)	1/2	0.00904(9)	0.8225
c (Å)	24.2266(15)	Sn01	”	”	”	”	0.1775
Volume (Å^3)	1412.41(16)	Sb02	0	0.16053(2)	0.31212(2)	0.00752(9)	0.8225
Density (calc.) (g cm^{-3})	7.268	Sn02	”	”	”	”	0.1775
R_{int}, Completeness	0.0147, 98.3%	Sb03	1	1/2	0.56561(2)	0.00781(9)	0.8225
R_1 $[I > 2\sigma(I)]$	0.0146	Sn03	”	”	”	”	0.1775
wR_2 $[I > 2\sigma(I)]$	0.0397						
Largest peak/hole (e Å^{-3})	1.252 / -1.032						
GOF / WGHT (A,B)	1.05 / (0, 5.3)						

TABLE S6. Abbreviated summary of supplementary crystal data, structural refinement, and atomic positions for Sb-rich $GdTi_3(Sb,Sn)_4$. Specific composition is $Gd_8Ti_{24}Sb_{26.98}Sn_{5.02}$ (corresponding to green data traces in main body). Complete CIF file is provided in the supplementary information. Specific (Sb,Sn) composition was set using results from energy dispersive spectroscopy. Preliminary neutron diffraction on $NdTi_3(Sb,Sn)_4$ suggests that there is no preferential occupation of Sn and no structural superlattice from potential (Sb,Sn) ordering. As such, we choose to provide CIF files as simple, homogenous alloys where (Sb,Sn) is shared among all anion sites.

Crystal Data and Refinement		Atomic Positions (fractional coordinates)					
Property	Value	Label	x	y	z	U_{iso}	Occ.
Formula	$\mathbf{Gd}_8\mathbf{Ti}_{24}\mathbf{Sb}_{26.98}\mathbf{Sn}_{5.02}$	Gd01	1	1/2	0.69527(2)	0.00701(7)	1
Space group, Z	Fmmm, 1	Ti01	1/2	1/2	0.59279(2)	0.00575(12)	1
a (Å)	5.7408(3)	Ti02	1/4	1/4	0.40343(2)	0.00578(10)	1
b (Å)	10.0524(5)	Sb01	1/2	0.32893(2)	1/2	0.00773(8)	0.8432
c (Å)	24.0511(11)	Sn01	”	”	”	”	0.1568
Volume (Å^3)	1387.96(12)	Sb02	0	0.15948(2)	0.31041(2)	0.00652(7)	0.8433
Density (calc.) (g cm^{-3})	7.523	Sn02	”	”	”	”	0.1567
R_{int}, Completeness	0.0110, 98.3%	Sb03	1	1/2	0.56677(2)	0.00652(8)	0.8432
R_1 $[I > 2\sigma(I)]$	0.0109	Sn03	”	”	”	”	0.1568
wR_2 $[I > 2\sigma(I)]$	0.0321						
Largest peak/hole (e Å^{-3})	1.123 / -0.843						
GOF / WGHT (A,B)	1.03 / (0, 2.7)						

TABLE S7. Single-crystal neutron diffraction results for a crystal of Sn-rich $NdTi_3(Sb,Sn)_4$, refined as $NdTi_3Sb_{3.241}Sn_{0.695}$, and modeled using a homogenous distribution of Sn across the Sb sites. Data was collected at 300 K. A comparison with other Sn distributions follows, but we have selected the homogenous distribution as the conservative model. Single-crystal neutron diffraction data were collected on the TOPAZ beamline at the Spallation Neutron Source, Oak Ridge National Laboratory [3]. Data collection strategy was planned with NeuXtalViz [1]. Peak integration employed the multiresolution machine-learning method of Peterson *et al.* [2]. Data reduction and normalization followed established TOPAZ protocols [3], including Lorentz, time-of-flight spectrum, detector efficiency, and absorption corrections. Intensities were exported in SHELX HKLF2 format,[4] with the wavelength retained for each reflection. Structure solution and refinement were performed using JANA2020 [5].

Chemical formula	$NdTi_3Sb_{3.241}Sn_{0.695}$
Formula weight	765
Crystal system	Orthorhombic
Space group	Fmmm
Temperature (K)	300
a (Å)	5.7642(9)
b (Å)	10.1273(19)
c (Å)	24.270(4)
V (Å^3)	1416.8(4)
Z	8
Radiation	Neutron ($\lambda = 0.4$–3.5 Å)
Absorption coefficient	$\mu = 0.02576 + 0.02654\lambda$ mm^{-1}
Crystal size (mm)	$0.2 \times 2 \times 4.5$
Diffractometer	TOPAZ
Measured reflections	5107
Independent reflections	4975
Observed reflections [$I > 3\sigma(I)$]	4964
R_{wp}, R_1, wR_2	0.036, 0.048, 0.114
Goodness of fit	1.27
Refined parameters	42
$\Delta\rho_{\max}$, $\Delta\rho_{\min}$ (fm Å^{-3})	0.55, -0.79

Atom	x	y	z	Occ.	Site	Sym.
Nd1	0	0	0.304860(10)	1	8i	mm2
Ti1	0.25	0.25	0.095631(17)	1	16j	..2
Ti2	0	0	0.09220(2)	1	8i	mm2
Sb1	0	0.16057(3)	0.187835(11)	0.794(16)	16m	m..
Sn1′	0	0.16057(3)	0.187835(11)	0.174(16)	16m	m..
Sb2	0	0	0.434459(14)	0.826(16)	16m	mm2
Sn2′	0	0	0.434459(14)	0.174(16)	16m	mm2
Sb3	0	0.17074(4)	0	0.826(16)	8i	m2m
Sn3′	0	0.17074(4)	0	0.174(16)	8i	m2m

Atom	U_{11}	U_{22}	U_{33}	U_{12}	U_{13}	U_{23}
Nd1	0.00475(9)	0.00567(13)	0.00438(11)	0	0	0
Ti1	0.00463(14)	0.00433(19)	0.00523(18)	$-0.00023(9)$	0	0
Ti2	0.00494(19)	0.0041(3)	0.0052(2)	0	0	0
Sb1	0.00597(10)	0.00494(13)	0.00405(11)	0	0	0.00035(7)
Sn1′	0.00597(10)	0.00494(13)	0.00405(11)	0	0	0.00035(7)
Sb2	0.00555(13)	0.00529(18)	0.00599(16)	0	0	0
Sn2′	0.00555(13)	0.00529(18)	0.00599(16)	0	0	0
Sb3	0.00833(14)	0.00727(18)	0.00208(14)	0	0	0
Sn3′	0.00833(14)	0.00727(18)	0.00208(14)	0	0	0

TABLE S8. Brief summary of preliminary neutron diffraction refinements on Sn-rich $NdTi_3(Sb,Sn)_4$. The table compares models with no Sn, equal Sn distribution, and freely refined Sn occupation. The corresponding observed versus calculated intensity plot is shown below for the equal-distribution model.

	$NdTi_3Sb_4$ (No Sn)	$NdTi_3(Sb,Sn)_4$ (homogenous Sn)	$NdTi_3(Sb,Sn)_4$ (Preferential Sn)
R_{wp}	3.67	3.62	3.61
wR_2	11.09	10.94	10.85
Composition type	Fixed	Refined	Refined
Composition	$NdTi_3Sb_4$	$NdTi_3Sb_{3.241}Sn_{0.695}$	$NdTi_3Sb_{3.261}Sn_{0.677}$

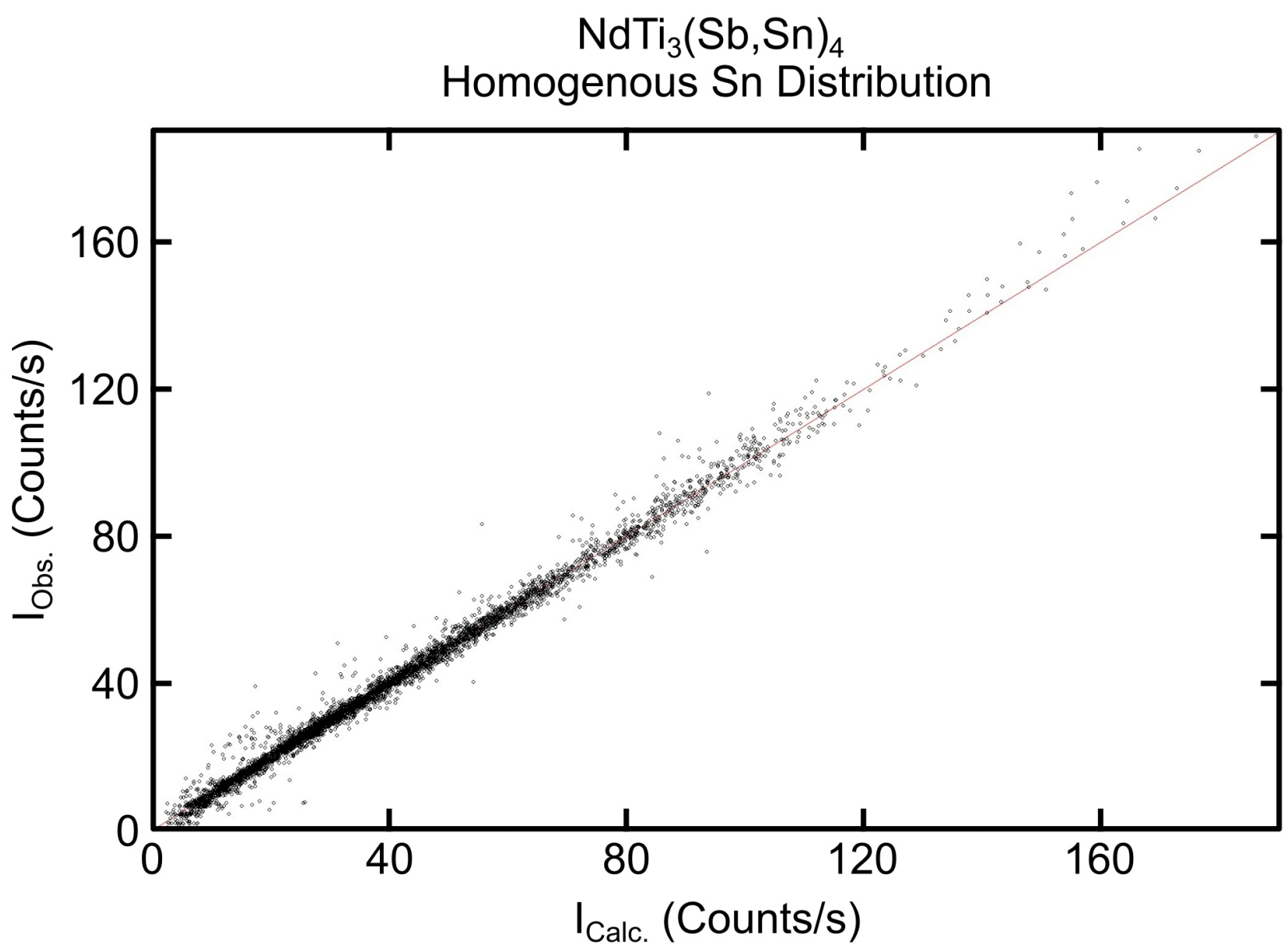

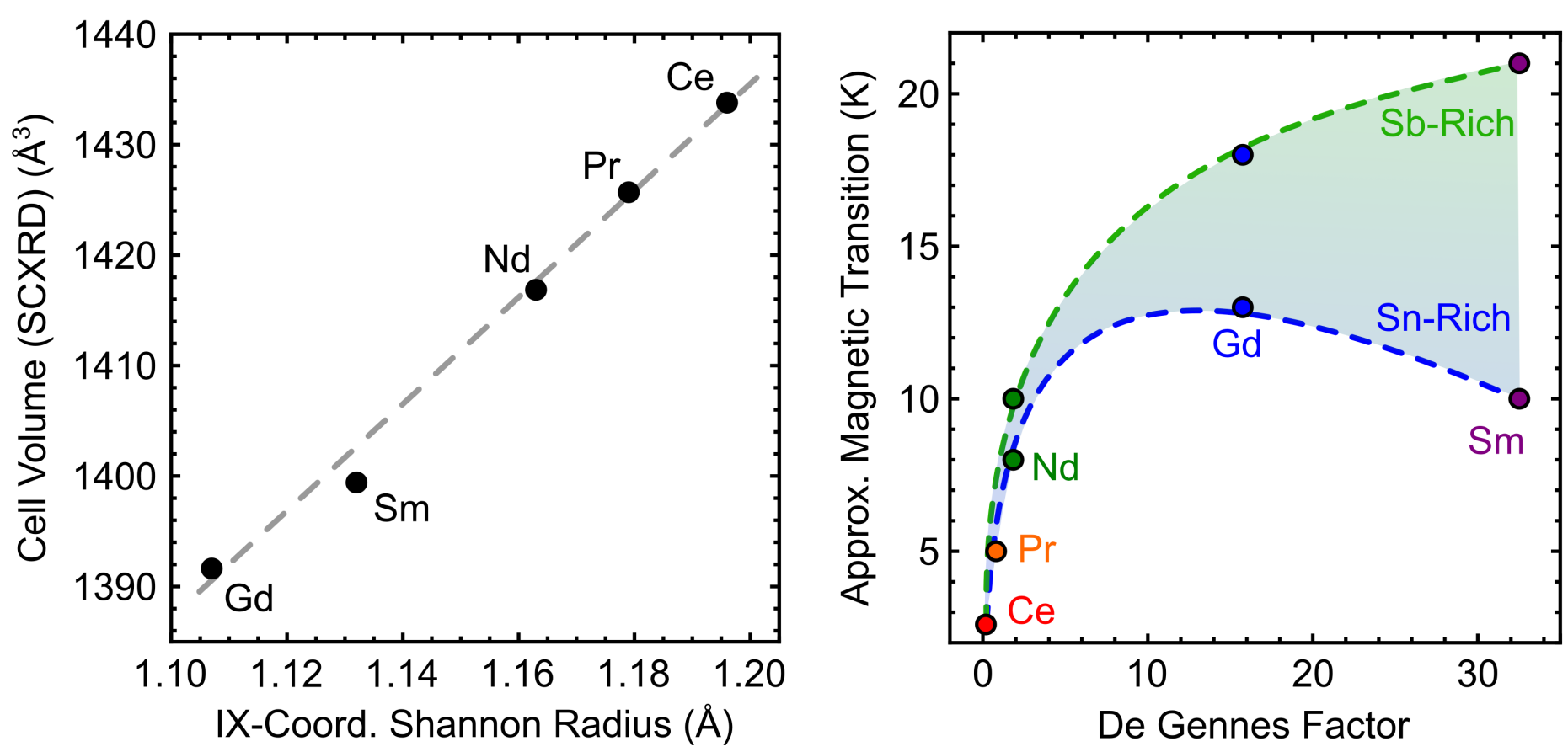

FIG. S4. Supplementary plots showing (a) the trend with lattice parameter throughout the rare-earth series (using only the Sn-rich end-member for all compounds), and (b) the De Gennes factor versus the magnetic transition temperatures, where both the Sn-rich and Sb-rich compounds are shown for each rare-earth.

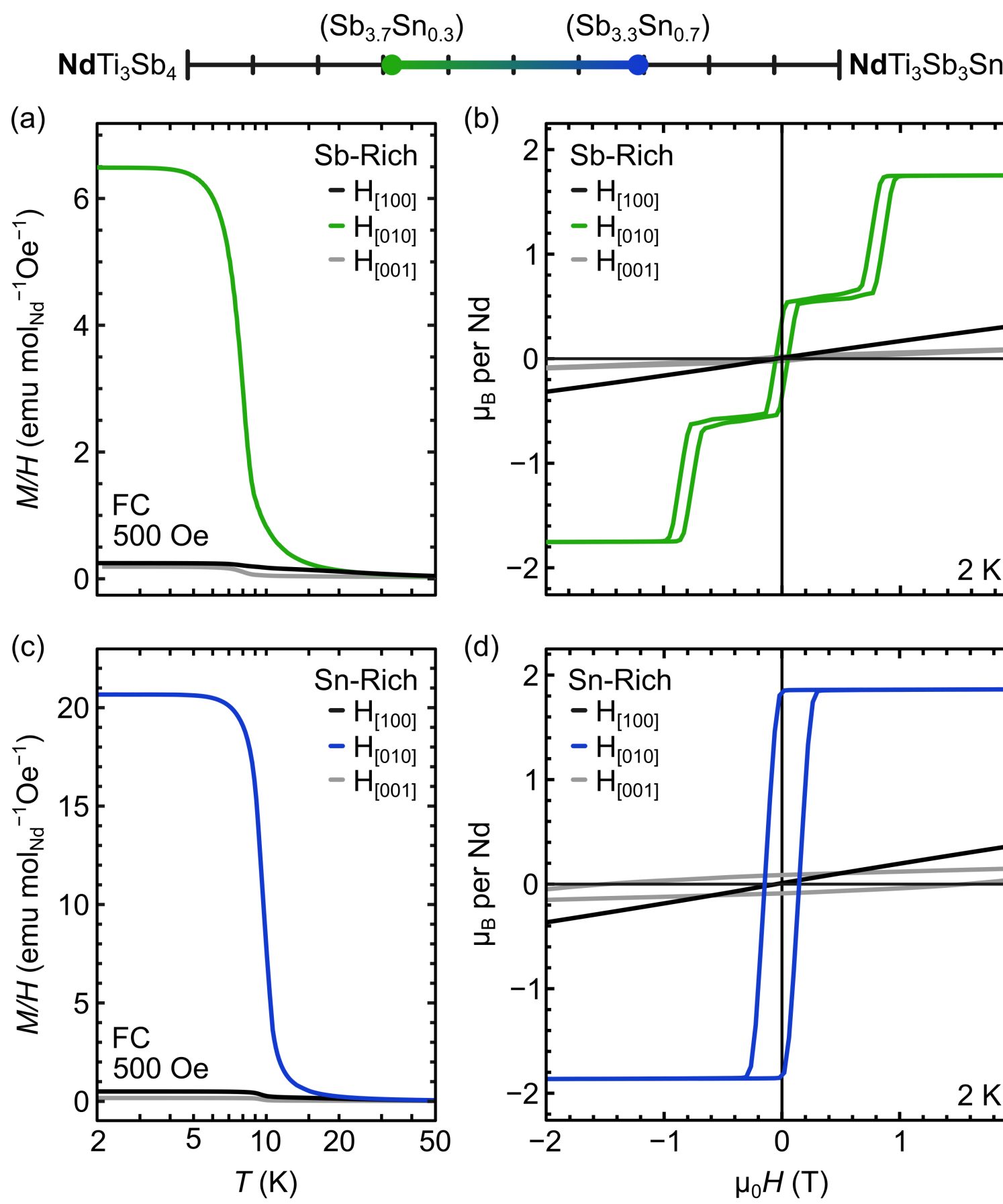

FIG. S5. Supplementary characterization data for $NdTi_3(Sb,Sn)_4$ alloy series. The Nd-containing series shows properties remarkably reminiscent of the $SmTi_3(Sb,Sn)_4$ data shown previously. Temperature-dependent magnetization for Sb-rich $NdTi_3(Sb,Sn)_4$ (a) shows a broad peak with a plateau which is weaker than would be expected for a prototypical FM. Isothermal magnetization results (b) reveal field-induced metamagnetic transitions with small but appreciable hysteresis in both transitions, reminiscent of the A(FM) state proposed for Sb-rich $SmTi_3(Sb,Sn)_4$. Sn-rich $NdTi_3(Sb,Sn)_4$ resembles a more prototypical FM with onset of magnetic order around 10 K in temperature-dependent magnetization measurements (c). Isothermal magnetization (d) confirms a FM response with a relatively weak coercive field less than 0.2 T. Note that $NdTi_3(Sb,Sn)_4$ exhibits an easy-axis along *b*.

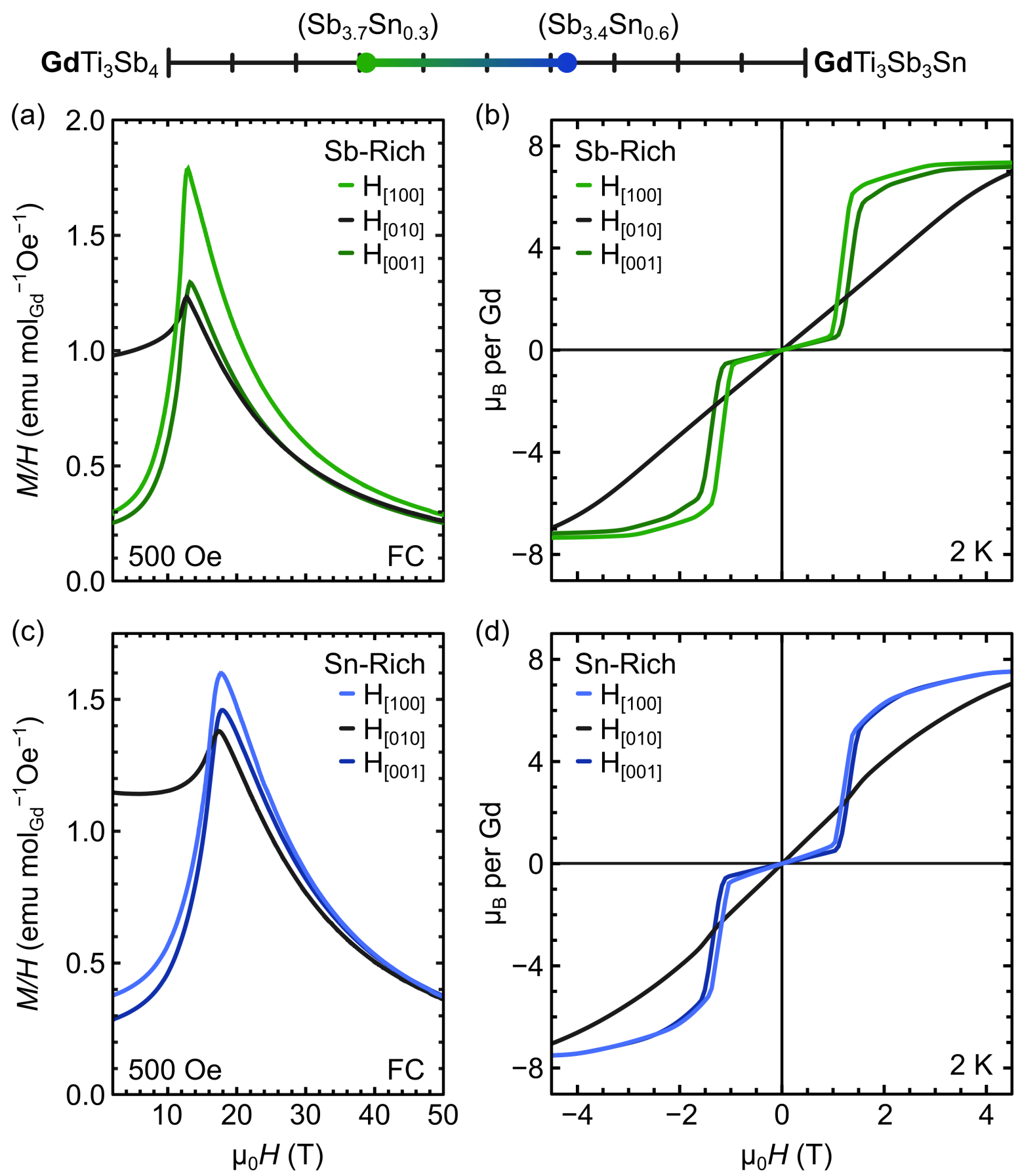

FIG. S6. Supplementary characterization data for $GdTi_3(Sb,Sn)_4$ alloy series. The Gd-containing series shows properties consistent with the bismuthide $GdTi_3Bi_4$ published previously. Despite the substantial (Sb,Sn) solubility range in $GdTi_3(Sb,Sn)_4$, the system is substantially less responsive to the doping, potentially due to the large spin-only moment of Gd^{3+}. Both the Sb-rich (a) and Sn-rich (c) temperature-dependent magnetization exhibit onset of AFM order around 13 K and 18 K, respectively. Both isothermal magnetization curves (b,d) are remarkably similar and plateau to values expected for the full Gd^{3+} moment. A clear field-induced spin-flop transition replete with a gradual spin reorientation is observed. Field-induced metamagnetism in the $GdTi_3(Sb,Sn)_4$ series occurs for fields directed along either *c* or *a*, consistent with $GdTi_3Bi_4$. These results suggest that this compound may have a non-collinear AFM structure or other complex anisotropy.

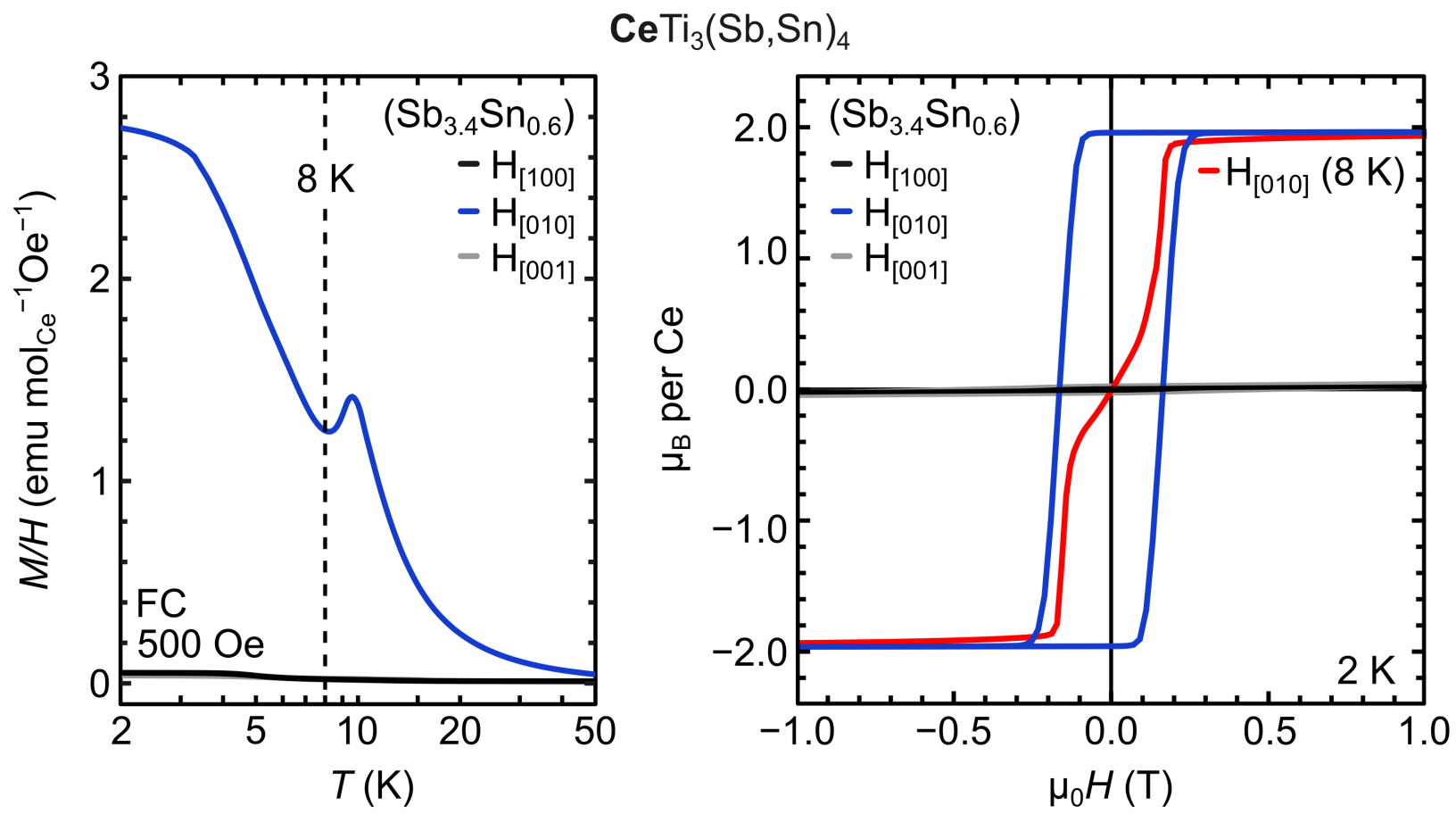

FIG. S7. Supplementary characterization data for $CeTi_3(Sb,Sn)_4$ alloy series. The Ce-containing compound exhibits an AFM cusp at 10 K followed by a FM-like (easy *b*-axis) response that saturates upon cooling below 3 K. Properties are broadly consistent with the magnetization behavior seen in the Sn-rich $SmTi_3(Sb,Sn)_4$ series. Field-dependent measurements at 8 K confirm AFM (metamagnetic) nature of the 10 K peak. As in Sn-rich $SmTi_3(Sb,Sn)_4$, the AFM response appears to be suppressed and replaced with FM order by 2 K.

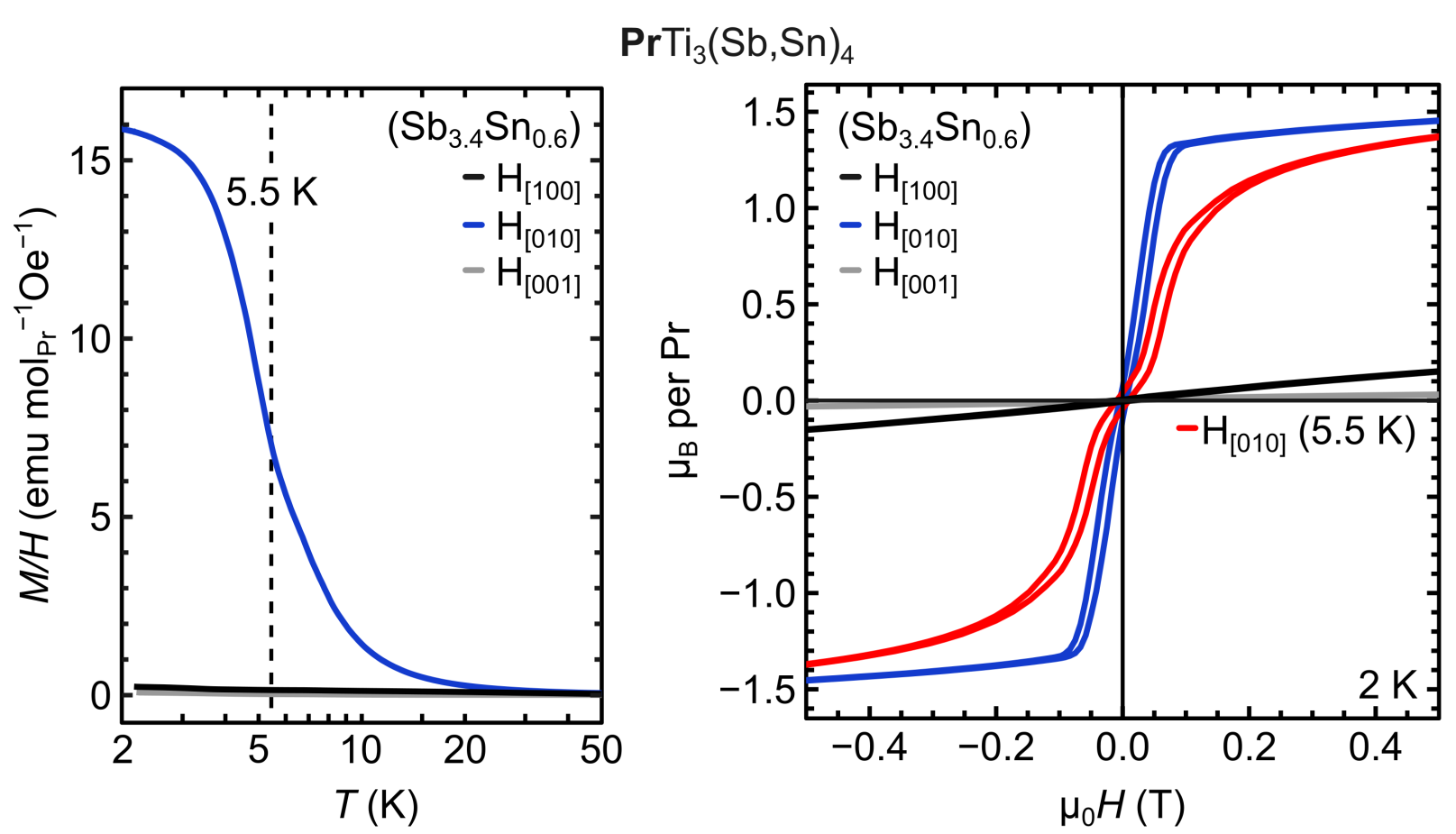

FIG. S8. Supplementary characterization data for $PrTi_3(Sb,Sn)_4$ alloy series. The Pr-containing compound exhibits a broad magnetization rise below approximately 5.5 K. Perhaps $PrTi_3(Sb,Sn)_4$ exhibits features similar to the rest of the rare-earth compounds (e.g., a broad AFM shoulder followed by a FM rise with easy *b*-axis magnetization), but the details are not clear. The case of Pr is further complicated by the known sensitivity of the Pr-ion to local disorder and CEF effects.

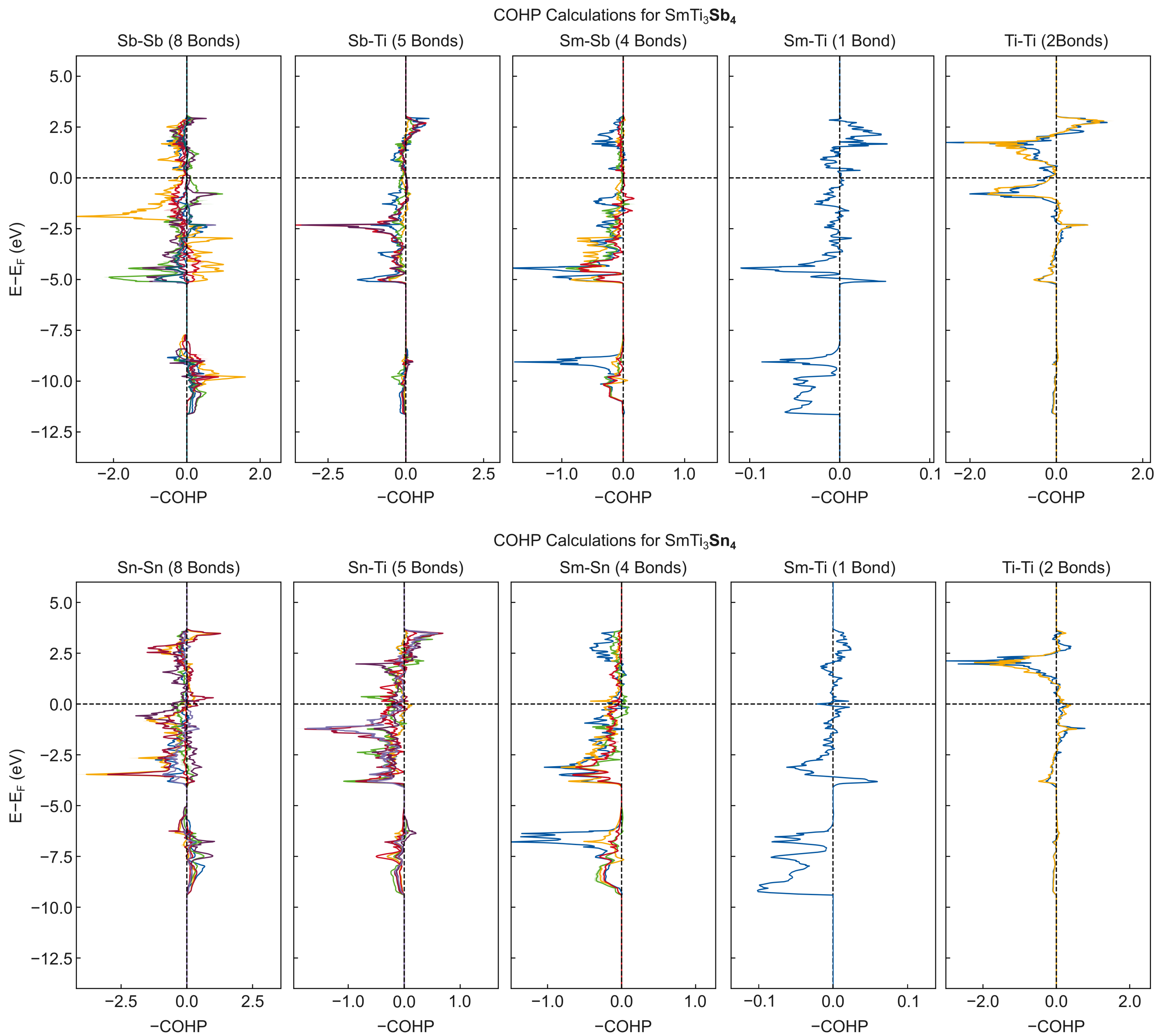

FIG. S9. Supplementary Crystal Orbital Hamilton Population (COHP) data for both $SmTi_3Sb_4$ (top 5 panels) and $SmTi_3Sn_4$ (bottom 5 panels). In the main body we focused on the Ti-Ti, Sb-Sb, and Sn-Sn interactions, but here we show all 5 types of interactions.

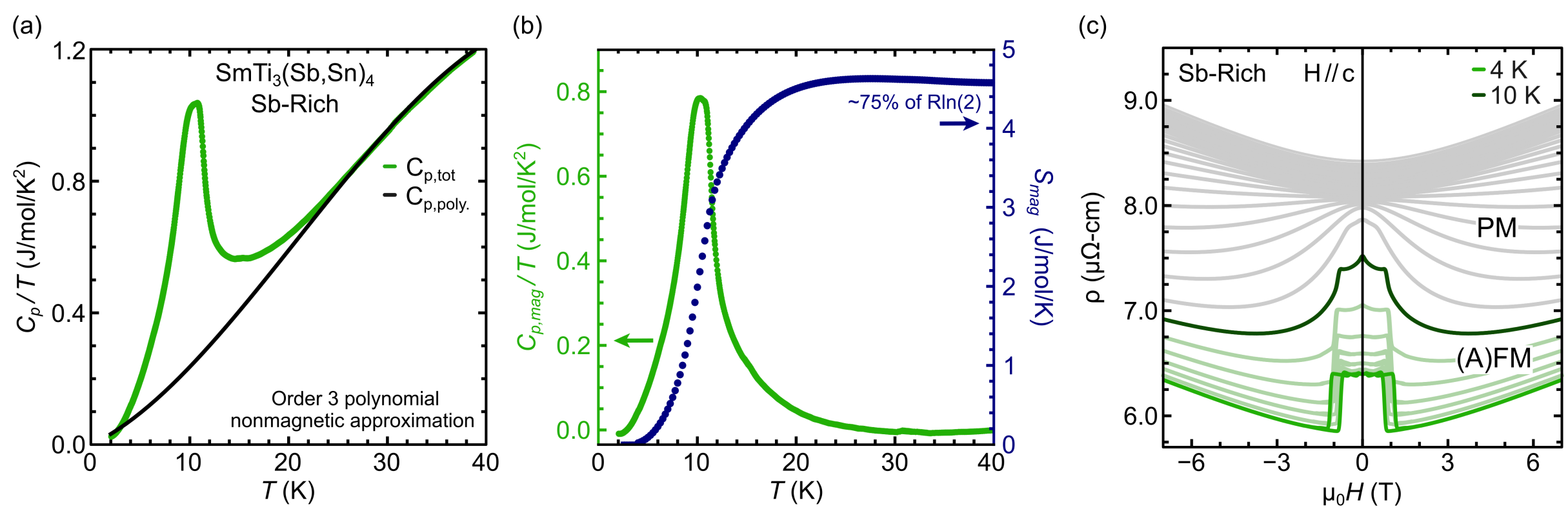

FIG. S10. Supplementary heat capacity data (a) showing the third-order polynomial used to approximate the nonmagnetic portion of the heat capacity. The polynomial is fit to the presumably nonmagnetic signal from 1.8–4 K and 22-40 K. The resulting magnetic heat capacity and integrated magnetic entropy are shown in (b), demonstrating that the approximated entropy release is approximately 75% of Rln(2). Supplementary magnetoresistance data (c) showing the same data as in the main body, but with no vertical shift applied to the light green data.

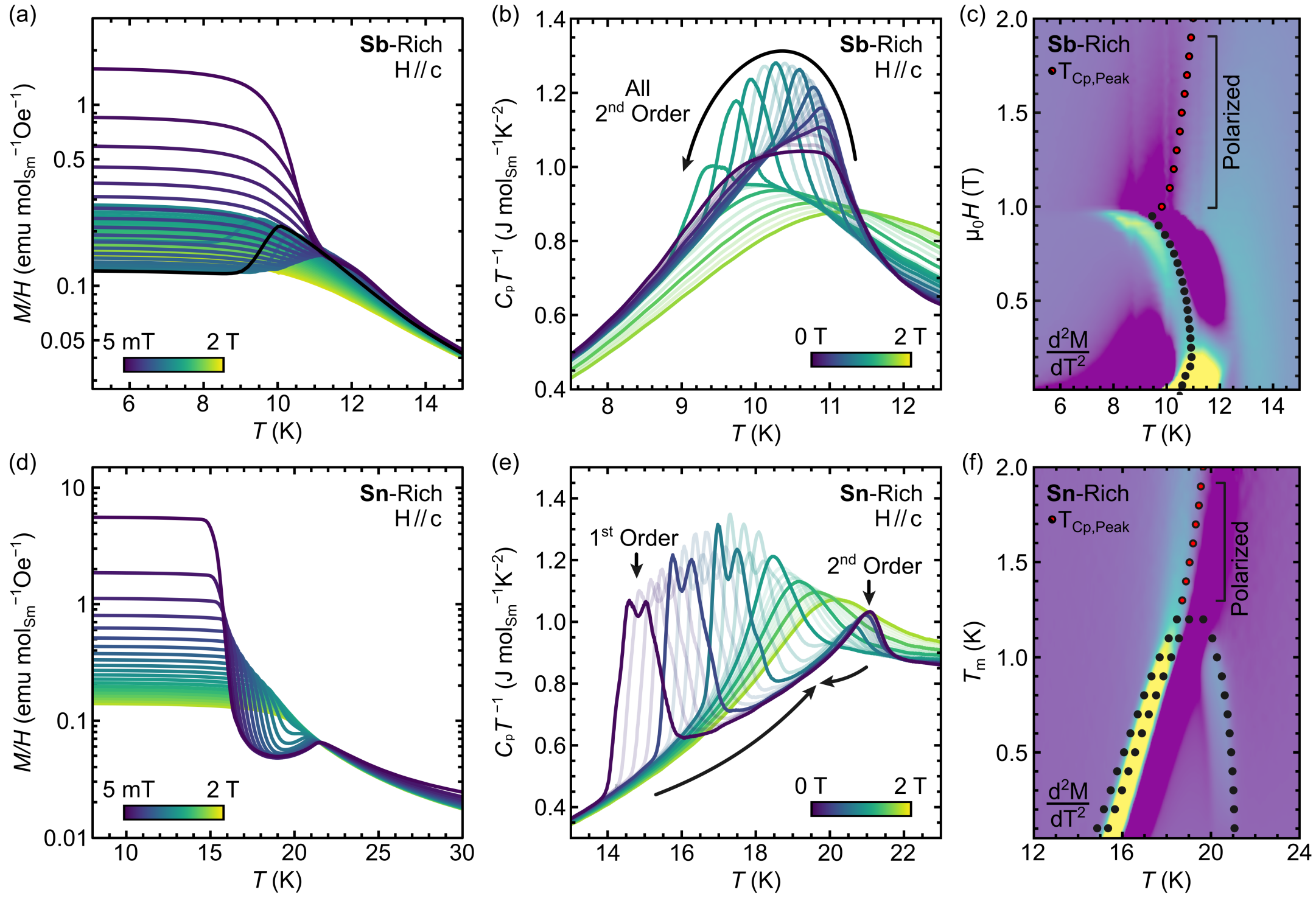

FIG. S11. Supplementary field-dependent measurements for the Sb-rich and Sn-rich $SmTi_3(Sb,Sn)_4$ series. (a,d) Temperature-dependent magnetization curves for the Sn-rich and Sb-rich termini under various magnetic fields. The AFM-like cusp in the Sn-rich sample is evident and appears gradually suppressed under applied field. The AFM-like cusp in the Sb-rich sample is more subtle at low fields, but is strengthened and more dominant at intermediate fields (highlighted in black for clarity). (b,e) Field-dependent large-pulse heat capacity measurements to help visualize the multiple heat capacity peaks and their interplay with fields. (c,f) Composite plots where the gradient background is the second-derivative of the field- and temperature-dependent magnetization. Solid points are identified from the field- and temperature-dependent heat capacity results.

Here we provide more discussion and context around the field-dependent heat capacity and magnetization results for Sb-rich and Sn-rich $SmTi_3(Sb,Sn)_4$:

Figure S11(a-f) examine the field-dependence of the Sb-rich and Sn-rich endpoints. This is particularly instructive when examining the heat capacity results, as we can clearly see the multiple peak structure of the results. We will examine both the magnetization and heat capacity results to help provide a more exhaustive interpretation of the results. Figure S11(a,d) examines the field-dependence of the magnetization curves with a logarithmic y-axis. For the Sn-rich sample (d) The higher-temperature AFM feature is clearly visible for a relatively wide range of fields, though it is progressively suppressed with the application of fields. For the Sb-rich sample (a) the situation is a bit more complex. At low fields there is a clear shoulder at higher temperatures (≈12 K) indicative of the onset of AFM-dominated interactions. However, unlike the Sn-rich sample, the application of a higher magnetic field does not progressively suppress the AFM interaction. Instead, there is an intermediate regime (note the black curve) where the AFM-like cusp is the dominant feature in the magnetization. This is consistent with the step-like features in the Sb-rich magnetization observed in the main body, as we know that intermediate fields at approximately 10 K should be consistent with a magnetization response that resembles a prototypical spin-flop AFM.

The heat-capacity curves are perhaps the most useful way to exhibit the interplay between the FM and AFM interactions in the system. Figure S11(e) shows the field-dependent (H||c) heat capacity results for the Sn-rich terminus. The purple (zero-field) curve clearly shows the split first-order peak and the higher-temperature AFM-like second-order peak. We've shown the series of field-dependent measurements with every 5th curve highlighted for clarity. At small to moderate fields (0-1 T) the first-order FM peak is affected the most, shifting towards higher temperatures while the AFM peak is only slightly broadened/shifted to lower temperatures. Above 1 T the AFM peak is rapidly suppressed and shifted downwards, merging into the first-order peak, which has also broadened enough to lose the double peak structure. As suggested by all previous measurements, this indicates that the Sn-rich sample exhibits a higher-temperature AFM which competes (and loses) to a FM-dominated ground state.

Now we turn to the more complex Sb-rich $SmTi_3(Sb,Sn)_4$ field-dependent heat capacity in Figure S11(e). The zero-field (purple) curve is clearly broad, with a clear right and left shoulder. With small applied fields (up to about 0.2 T) the centroid of the peak shifts towards higher temperatures (FM-dominant), the right shoulder sharpens into a well-defined peak, and the left shoulder is suppressed/merged. Above 0.2 T the sharpened peak begins to shift towards lower temperatures (AFM-dominant). Close to $\approx$1 T, the peak quickly broadens into a double-peak feature right before the entire feature is suppressed in the field-polarized state. Again, we see the dance between AFM and FM interactions indicative of the $SmTi_3(Sb,Sn)_4$ family, and the much more intertwined nature of the A(FM) ground state.

Figure S11(c,f) summarizes the field-dependent magnetization and field-dependent heat capacity results as field-temperature phase diagrams. The background color contour is produced through the second-derivative of the magnetization curves, while the discrete points are derived from the field-dependent heat capacity results.

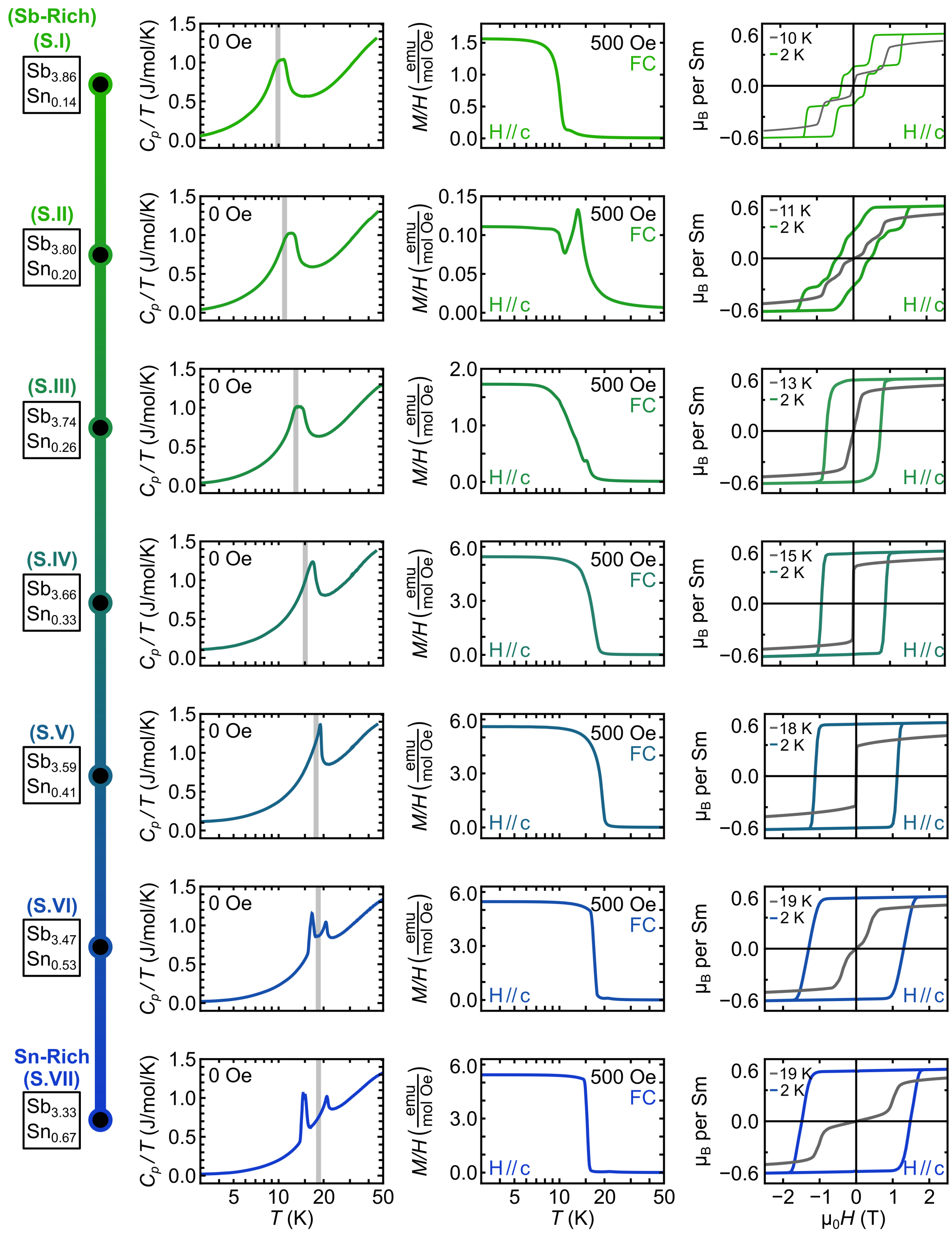

FIG. S12. Supplementary characterization data for the full series of samples traversing the $SmTi_3(Sb,Sn)_4$ solid solution to demonstrate the gradual shift in magnetic and thermodynamic properties. This is identical data to that shown in the main body, but separated onto individual axes for quantitative comparison. Shown from left to right are: (1) the measured (Sb,Sn) content of the single crystals, (2) a plot showing the temperature-dependent magnetization (3) zero-field heat capacity results, and (4) the corresponding isothermal magnetization curves at base temperature and an intermediate temperature (gray) intended to demarcate the transition between the two heat capacity features (where possible). For clarity, the intermediate temperature (gray) is also marked on the temperature-dependent magnetization.

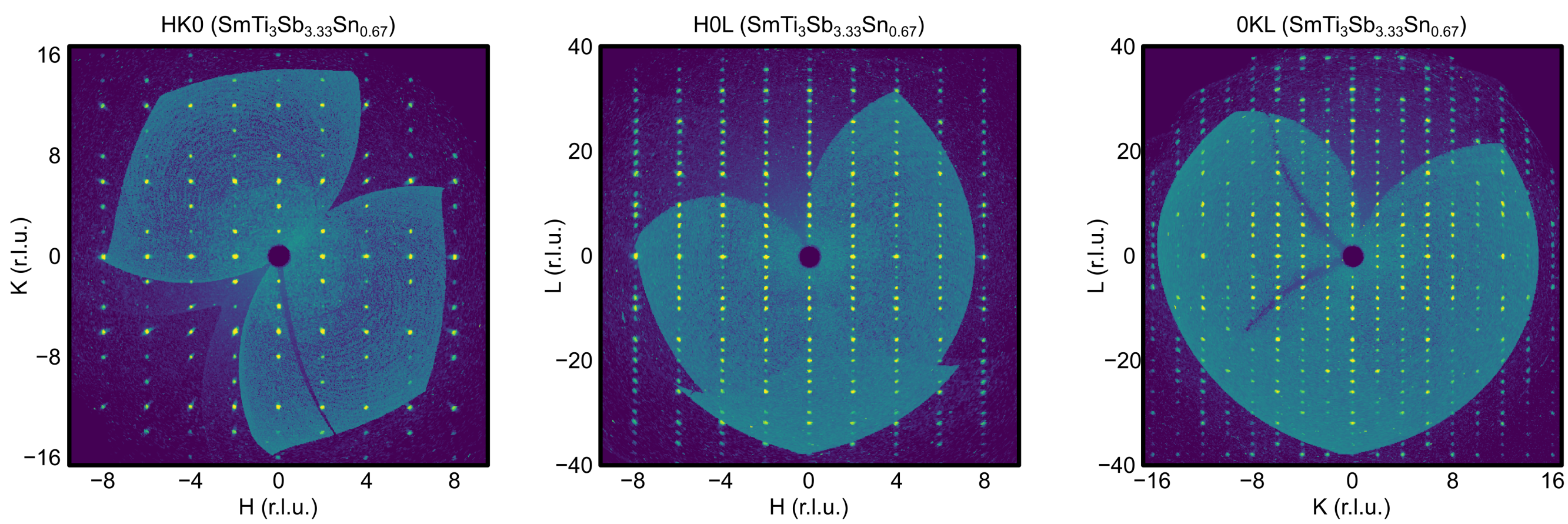

FIG. S13. Supplementary single-crystal X-ray diffraction precession images for Sn-rich $SmTi_3(Sb,Sn)_4$. Images are shown for HK0, H0L, and 0KL. Crystals prepared *via* exfoliation (please see methods section for details) have excellent mosaic and show no evidence of twinning. Due to the required sample processing, crystals are extremely thin with exaggerated dimensions (e.g., $0.196\times0.143\times0.002$ mm). As such absorption corrections and considerations for weak peak statistics must be kept in mind.

[1] Z. Morgan, Z. Xiao, S. Pawledzio, I. Ye, S. Jin, V. E. Lynch, T. Proffen, C. M. Hoffmann, and X. Wang, *J. Appl. Crystallogr.*, manuscript in preparation.
[2] V. Reshniak, X. Wang, G. Zhang, S. Liu, and J. Yin, arXiv:2406.05133 (2024).
[3] A. J. Schultz, M. R. V. Jorgensen, X. P. Wang, R. L. Mikkelson, D. J. Mikkelson, V. E. Lynch, P. F. Peterson, M. L. Green, and C. M. Hoffmann, *J. Appl. Crystallogr.* **47**, 915 (2014).
[4] G. M. Sheldrick, *Acta Crystallogr. C* **71**, 3 (2015).
[5] V. Petříček, L. Palatinus, J. Plášil, and M. Dušek, *Z. Kristallogr. Cryst. Mater.* **238**, 271 (2023).
